# Synthesis of 4-(2-fluorophenyl)-7-methoxycoumarin: experimental and computational evidence for intramolecular and intermolecular C–F···H–C bonds

**DOI:** 10.3762/bjoc.16.22

**Published:** 2020-02-10

**Authors:** Vuyisa Mzozoyana, Fanie R van Heerden, Craig Grimmer

**Affiliations:** 1 School of Chemistry and Physics, University of KwaZulu-Natal, Private Bag X01, Scottsville, 3209, Pietermaritzburg, South Africa

**Keywords:** DFT, F···H hydrogen bond, fluorinated phenylcoumarin, Pechmann reaction, through-space coupling

## Abstract

4-(2-Fluorophenyl)-7-methoxycoumarin (**6**) was synthesized by Pechmann reaction under mild conditions via a three-step reaction. The solution-state ^1^H NMR spectra of **6** showed a strong intramolecular interaction between F and H5 (*J*_FH_ = 2.6 Hz) and ^13^C NMR suggested that this C–F···H–C coupling is a through-space interaction. The 2D ^19^F-{^1^H} HOESY and ^1^H-{^19^F} 1D experiments were done to confirm this F···H interaction. The single crystal X-ray structure and the DFT-optimized structure showed that the fluorinated phenyl ring favors the orientation with the fluorine atom closer to H5 than H3. The X-ray structure also showed the existence of the intermolecular C–F···H–C interaction.

## Introduction

Coumarins constitute one of the big classes of naturally occurring compounds. The first coumarin was isolated from the tonka bean (Dipteryx odorata) in 1820 and, to date, more than 1300 coumarins have been identified from natural sources [1–2]. Coumarins have been reported to play a vital role as food and cosmetics constituents, cigarette additives, and dye-sensitized solar cells [3–4]. In addition, coumarins possess some biological activities such as anti-inflammatory [5], antitumor [6], anti-oxidant [7], antibacterial [8], hepatoprotective, anticoagulant, antiviral and antithrombotic activities [9]. The variety of uses of these compounds resulted in an increase in demand for large quantities of coumarins. Due to an insufficient natural supply to meet this demand for these compounds, numerous methods for the synthesis of these compounds have been developed, examples are the Pechmann condensation [10–11], Stille coupling reaction [12], Knoevenagel condensation [13], Heck coupling reaction [14], Kostanecki reaction, Baylis–Hillman reaction [15], Michael reaction [16], Suzuki–Miyaura cross-coupling reaction [17], Negishi cross-coupling reaction [18] and Wittig reaction [17]. The concept of the incorporation of fluorine into organic molecules has gained much interest since Fried and Sabo reported the improvement of the therapeutic index of cortisol by the incorporation of a fluorine atom in the 9α position of the structure [19]. Since then, the fluorine-containing drugs have come onto the market and they are amongst the best-selling pharmaceutical drugs, including Lipitor^®^, Prevacid^®^, Advair Discus^®^ and Lexapro^®^ [20–22]. The incorporation of fluorine may improve the activity of biologically active compounds as it imparts a variety of properties such as enhanced binding interaction, metabolic stability, and reaction selectivity by changing physical and chemical properties [23–26].

Hydrogen bonds (HBs) are associated with highly electronegative atoms (oxygen, nitrogen, fluorine) and have been observed to govern the conformational structure of some molecules as well as the alignment of the molecules within a crystal structure [27–29]. Moreover, HBs have been reported to play a vital role in a ligand–receptor interaction that determines the biological activity of a molecule. Oxygen and nitrogen have been proven to be good hydrogen-bond acceptors which form strong intermolecular and intramolecular hydrogen bonds, however, fluorine is still denied hydrogen-bond acceptor status by some scientists.

There is evidence of the existence of C–F···H interaction in organic molecules [30–31]. Early reports by Glusker and co-workers in 1983 and 1994 showed C–F···H interactions in structures found in the Cambridge Crystallographic Data Centre database [32]. Similar evidence was reported by Howard, O’Hagan, Desiraju and their co-workers where the C–F···H interaction was observed, although the conclusions of the two groups were different – O’Hagan et al. concluded that fluorine is not a good hydrogen-bond acceptor, whereas Desiraju et al. concluded that the interaction has genuine hydrogen-bond character [24,33–36].

The C–F···H–C interaction is amongst the weakest of hydrogen bonding phenomena because a carbon acid (C–H) is weak, therefore is a weak donor, and the acceptor is non-polarizable, therefore is a poor acceptor [34–36]. Wang and co-workers reported the existence of the C–F···H–C intramolecular hydrogen bond in the structure of aromatic triazole foldmers [37]. In their study, using crystallographic and DFT data, they concluded that their folded conformers are held by C–F···H–C hydrogen bonds. To further these studies, we have synthesized a fluorine-containing phenylcoumarin in order to study the fluorine-hydrogen bond. The crystal structure and solution-state NMR data of the coumarin **6** were studied to examine any C–F···H–C hydrogen bond interactions. DFT calculations were performed to determine the preferred conformations of the structure that might exhibit a C–F···H–C hydrogen bond.

## Results and Discussion

### Synthesis of 2-fluorophenylcoumarin **6**

**Scheme 1:**
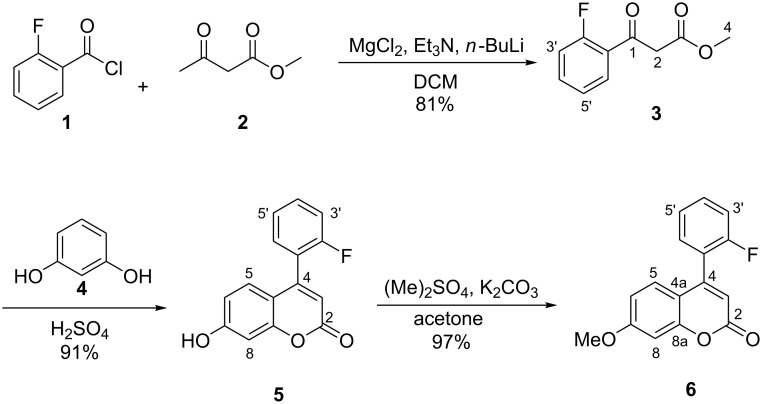
Synthesis of 4-(2-fluorophenyl)-7-methoxycoumarin (**6**).

4-(2-Fluorophenyl)-7-methoxycoumarin (**6**) was synthesized under mild conditions via a three-step reaction (Scheme 1) and the first step was the synthesis of the fluorinated β-keto ester **3**. Methyl acetoacetate (**2**) was treated with MgCl_2_, Et_3_N and *n*-BuLi in DCM and then with 2-fluorobenzoyl chloride (**1**) to yield methyl 2-fluorobenzoylacetate (**3**) [38–39]. These reactions are very rare in the literature, however, there are similar reactions for the synthesis of β-keto esters as reported by Sijbesma et al. [40] and Anwar [41]. The second step of the synthesis was the Pechmann reaction, commonly used for the synthesis of coumarins [42–43]. Methyl 2-fluorobenzoylacetate (**3**) was reacted with resorcinol (**4**) in the presence of H_2_SO_4_ at 35 °C, and 7-hydroxy-4-(2-fluorophenyl)coumarin (**5**) [39,44] was obtained as a light yellow solid. The last step of the synthesis was the methylation of the hydroxy group of coumarin **5** with dimethyl sulfate, to form 4-(2-fluorophenyl)-7-methoxycoumarin (**6**).

## Discussion

**Figure 1:**
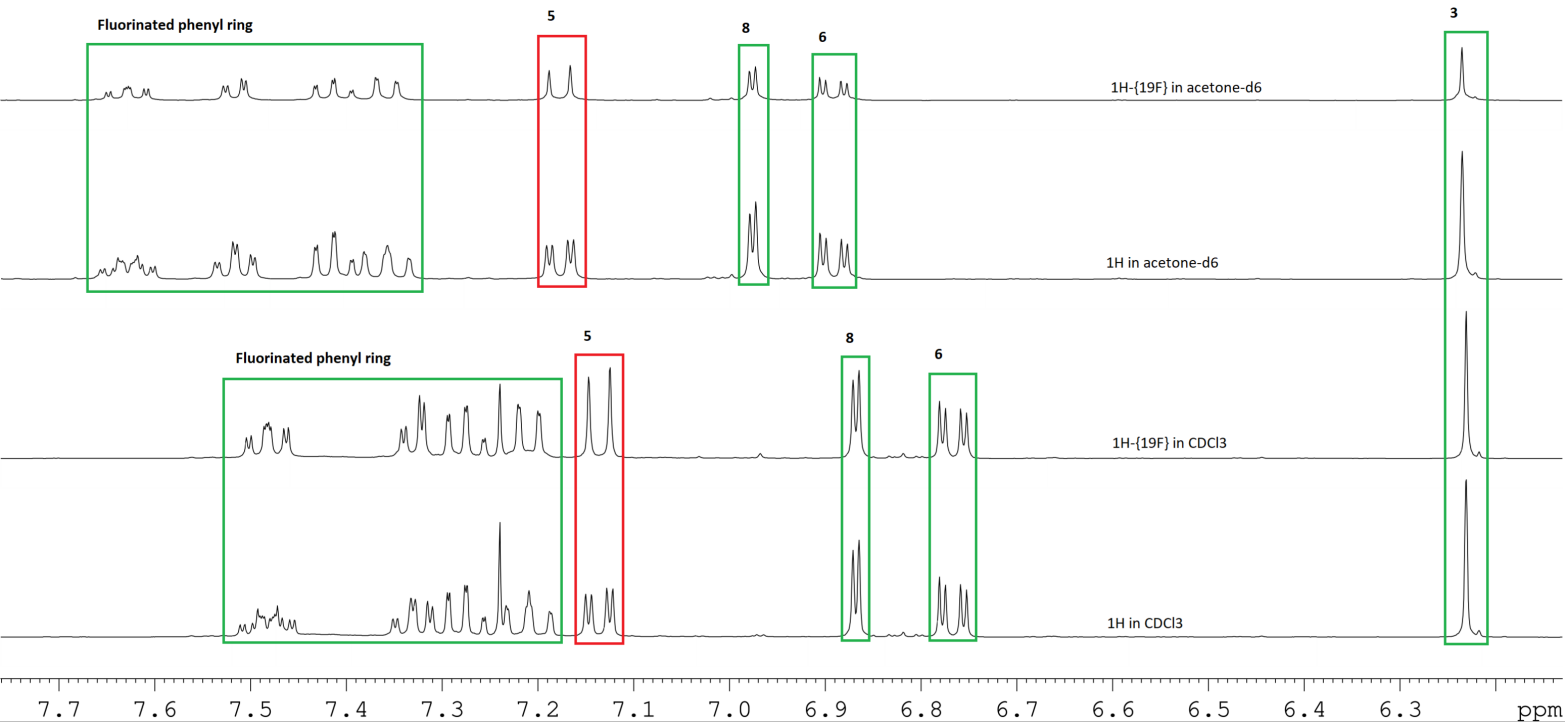
^1^H NMR spectra for the “aromatic” region of coumarin **6**; comparison of ^1^H spectrum and ^1^H-{^19^F} spectrum in CDCl_3_ and ^1^H spectrum and ^1^H-{^19^F} spectrum in acetone-*d*_6_.

During the synthesis of coumarin **6**, solution-state NMR spectroscopy was used to characterize compounds **3**, **5,** and **6** (^1^H and ^13^C spectra are available in Supporting Information File 1). The ^1^H spectrum of coumarin **6** showed H···F interactions for H3′, H4′, H5′ and H6′ which is typical through-bond (TB) coupling. However, the peaks that caught our particular attention were the singlet peak at 6.25 ppm and a doublet-of-doublets (dd) peak at 7.16 ppm assigned to H3 and H5, respectively (Figure 1). The H5 signal was expected to be a doublet (not a dd) due to ^3^*J* coupling to H6, since an H,H-COSY experiment does not show coupling between H5 and H8 (Figure S9, Supporting Information File 1). It became clear that the splitting of the signal from H5 was due to coupling with the ^19^F atom by comparing the spectra from the ^1^H and ^1^H-{^19^F} experiments (Figure 1) which showed the H5 peak as a doublet with ^19^F decoupling. While the doublet-of-doublets signal for H5 collapses into a doublet with ^19^F decoupling, there are no significant changes in the line-shape for the signal of H3 with ^19^F decoupling (Figure 1).

To determine whether the observation of ^19^F–H5 coupling for coumarin **6** was a solvent dependent phenomenon, a comparison was made between the ^1^H and ^1^H-{^19^F} spectra in CDCl_3_ and acetone-*d*_6_. Splitting of the H5 signal was observed in both solvents (Figure 1) suggesting that the ^19^F–H5 coupling for coumarin **6** is solvent independent. A literature report of the NMR characterization of a structurally similar 7-hydroxycoumarin performed in DMSO-*d*_6_ has the signal for H5 reported as a singlet [44].

Since coumarins **6** and **5** have similar structure, the only difference is at position seven, **6** has a methoxy group while **5** has a hydroxy group, a similar study was carried out for coumarin **5** (Figure S10b, Supporting Information File 1).

The question posed at this point was “is this a through-bond (TB) or through-space (TS) effect”?

**Figure 2:**
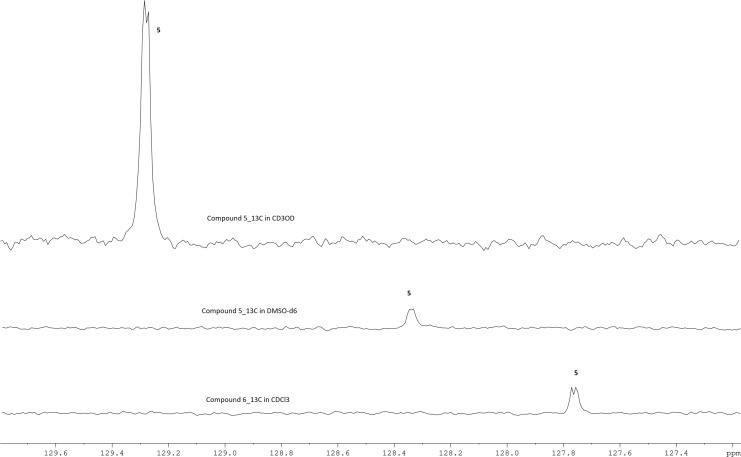
^13^C NMR spectra for coumarin **5** and **6**; showing the splitting of the signal corresponding to C5.

To answer this question, we analysed a ^13^C-{^1^H} spectrum of coumarin **6** dissolved in CDCl_3_ and the signal corresponding to C5 was found to be a doublet (*J* = 1.4 Hz) but the signals corresponding to C4 and C4a were singlets, and this indicates that this coupling is not a TB effect, because if it were a TB effect, the signals for C4 and C4a would also likely be split. Similar splitting of C5 was observed in coumarin **5**, also in different solvents as shown in Figure 2 and there was no splitting of the signals corresponding to C4 and C4a as shown in Figures S22 and S21 (Supporting Information File 1). These observations were not found in the similar derivatives of coumarin **5** (7-hydroxy-4-(3-fluorophenyl)coumarin and 7-hydroxy-4-(4-fluorophenyl)coumarin) found in the literature where the fluorine atom is in the third (C3') and the fourth (C4') position, respectively, of the phenyl ring [39]. The spectra for these derivatives showed an H5 signal as a doublet (not doublet-of-doublets as observed in coumarin **5** and **6**) and C5 as a singlet, indicating that they do not possess the through-space F···H5 or/and F···C5 coupling since the fluorine atom is a bit further away from H5 and C5.

**Figure 3:**
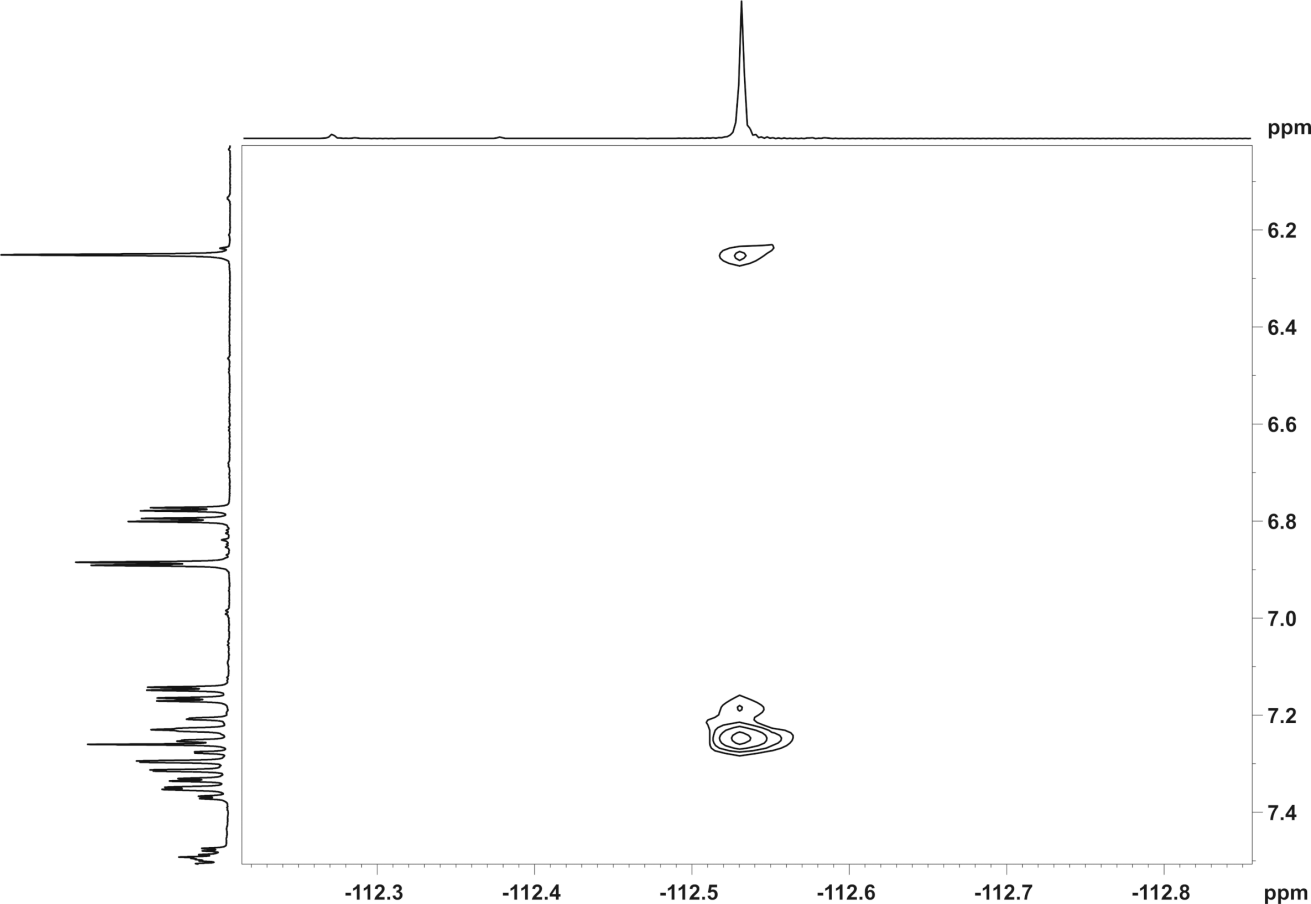
^19^F,^1^H-HOESY NMR spectrum for coumarin **6** illustrating two through-space interactions.

To confirm our findings, we further ran a ^19^F,^1^H-HOESY experiment and it showed clear H5···^19^F and H3···^19^F coupling (Figure 3). Evidence of a HOESY interaction between H5···^19^F and H3···^19^F indicates that neither the H3···^19^F nor the H5···^19^F interaction limits the C4–C1′ bond rotation.

**Figure 4:**
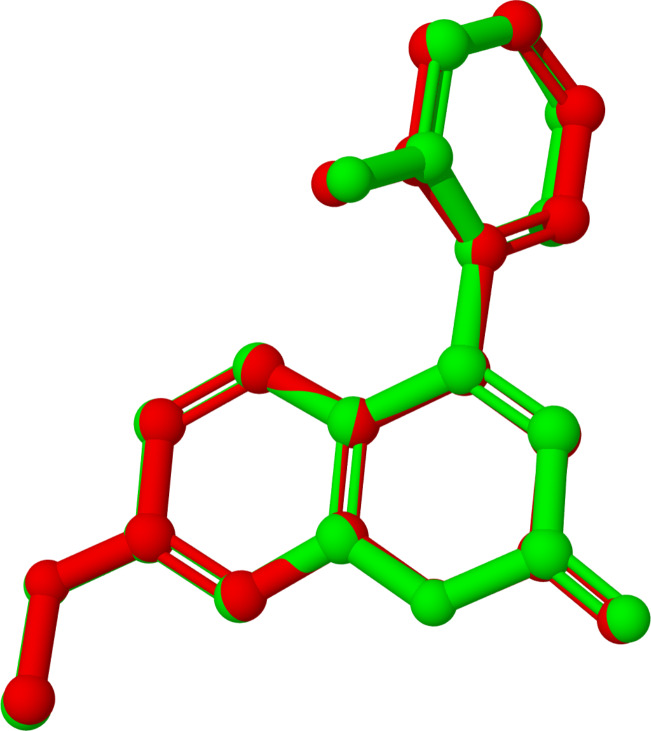
Superposition of single-crystal X-ray structure (red) and DFT-optimized structure (green); RMSD 0.3 Å (hydrogen atoms omitted for clarity).

The geometry of coumarin **6** (single molecule, gas phase) was optimized using the B3LYP functional and the 6-311G basis set, as implemented in Gaussian-09W (Rev. C.01) [45]. The superposition of the single-crystal X-ray structure (red) and the optimized structure (green) is shown in Figure 4.

**Figure 5:**
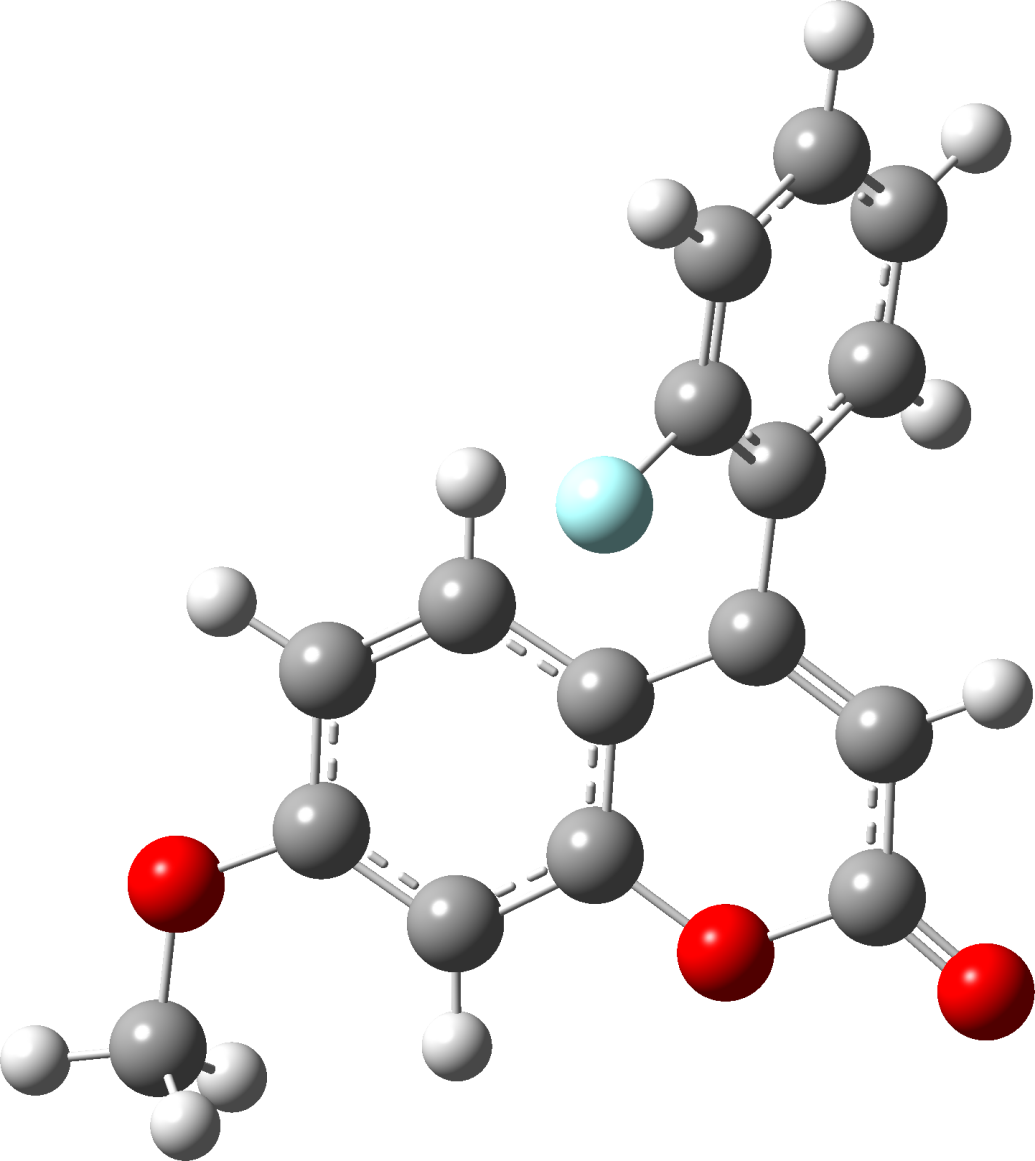
DFT-optimized structure for coumarin (**6**).

**Figure 6:**
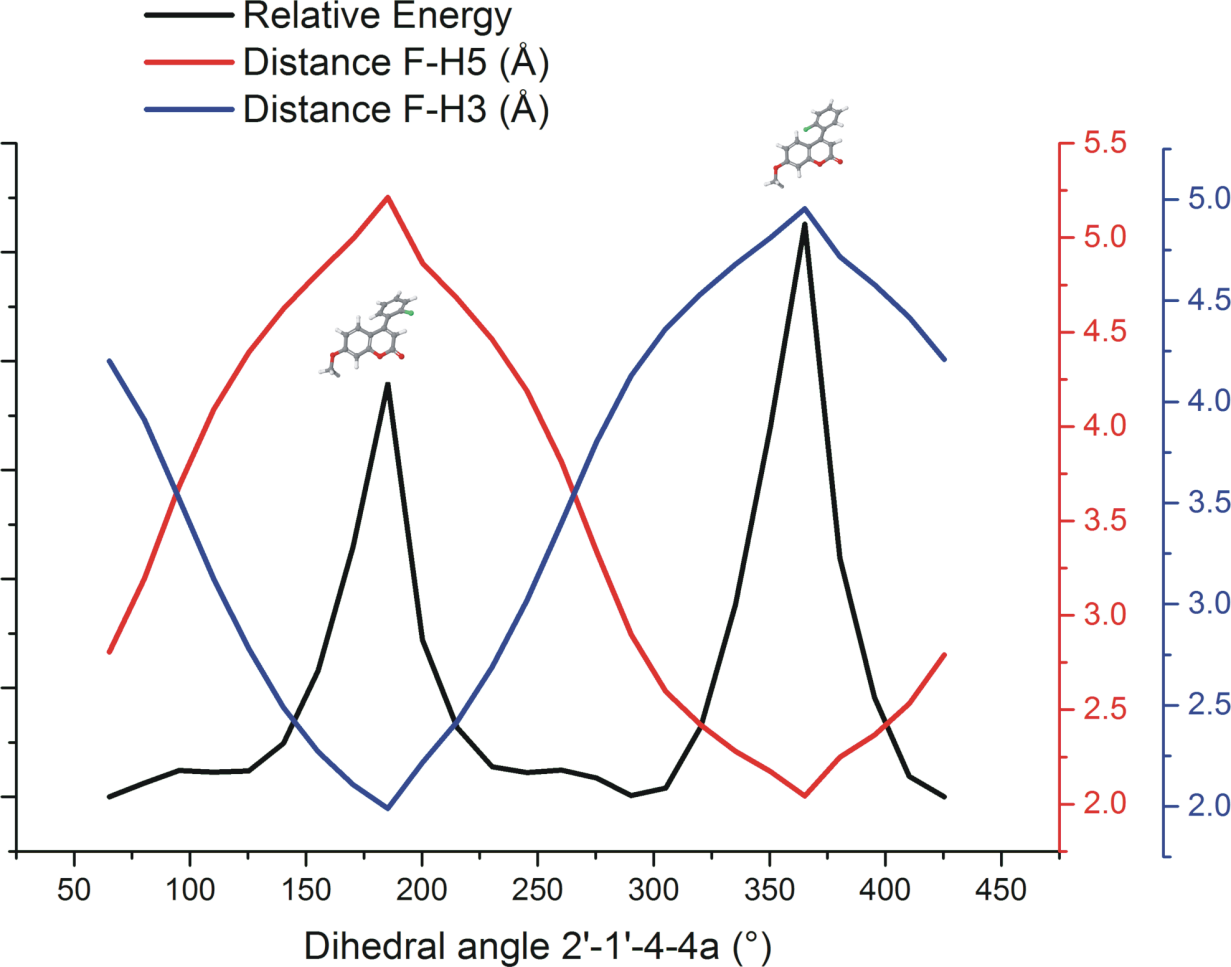
Plots of relative energy (black trace, no units), interatomic distance F–H5 (red trace, Å), interatomic distance F–H3 (blue trace, Å) as a function of dihedral angle Φ C2’–C1’–C4–C4a (°).

The DFT optimized geometric structure is shown in Figure 5 and has a dihedral angle, Φ (C2'–C1'–C4–C4a) of 65.3°. Following the optimization, the dihedral angle Φ was varied through a 360° rotation to examine the effect of changing the relative position of the fluorinated ring and the energy profile for this variation is shown in Figure 6. When Φ = 5°, the F···H5 distance is at its shortest (*d*_F_···_H5_ = 2.0 Å) and the fluorinated ring is almost coplanar with the coumarin ring, and the molecule is at its least stable conformation due to the electron–electron (e–e) repulsion of H5 and fluorine. The second least stable conformation is found at Φ *=* 185°, with the fluorine atom and H3 in close proximity (*d*_F_···_H3_ = 2.0 Å).

Examples of through-space coupling between fluorine and hydrogen atoms in organic molecules are reported in the chemical literature [46–47], with magnitudes as large as 7 Hz for ^7^*J*_HF_ [46]

One report of TS-coupling between F and H atoms comments that “*...it appears that significant coupling only occurs when the proton–fluorine closest approach distance is within the sum of the van der Waals radii of hydrogen and fluorine (c. 2.55 Å)...*” [47]. During rotation of the fluorophenyl ring (Figure 6), the F–H distance (for both F–H5 and F–H3) varies between 2 and 5 Å, so for some part of the rotation, both the distances F–H5 and F–H3 fall inside the limit of ≈2.55 Å, although not simultaneously. For a fixed geometry like that of the crystal structure, this would suggest that through-space F–H coupling would be observed between F and H5 but not between F and H3, as in Figure 1, where there is an obvious change to the shape of the signal corresponding to H5 but a negligible change to the shape of the signal corresponding to H3, with the application of {^19^F} decoupling. However, the F–H HOESY spectrum in Figure 3 shows coupling between the fluorine atom and both H5 and H3. The authors interpretation of these observations (Figure 1 and Figure 3) is that, in solution, rotation of the fluorophenyl ring (about C4–C1’) is permitted but that the average geometry of coumarin **6** has the fluorine atom closer to H5 than H3.

The theoretical NMR data for twenty-four conformations of coumarin **6** were obtained from Gaussian 09W (Rev C.01) at the B3LYP/6-311G level. Geometry optimization and calculation of NMR parameters for TMS and CCl_3_F at the same level provided reference chemical shifts for ^1^H, ^13^C, and ^19^F. The chemical shifts for the lowest energy structure (Φ = 65.3°) and the most unstable conformer (Φ = 5°) are used as examples (Table 1). The theoretical chemical shifts for the carbons appeared to be shifted downfield relative to the experimental carbon peaks (for both stable and unstable conformers) as shown by ‘change’ (Δ = −ve, experimental − theoretical) in Table 1.

**Table 1:** Experimental and theoretical (gas phase) ^1^H and ^13^C chemical shift (δ) for atoms within six bonds from fluorine for coumarin **6** and RSMD values.

Atom	Exp	Φ = 65.3° ^a^		Φ = 5° ^b^	
C/H	δ (ppm)	δ (ppm)	Δ(ppm)^c^	δ (ppm)	Δ(ppm)^c^

C2	160.9	169.1	−8.2	170.1	−9.2
C3	112.5	118.4	−5.9	117.7	−5.2
**H3**	**6.25**	**6.00**	**0.25**	**6.75**	**−0.50**
C4	150.5	158.0	−7.5	152.6	−2.1
C4a	112.4	120.8	−8.4	120.2	−7.8
C5	127.8	135.0	−7.2	139.2	−11.4
**H5**	**7.16**	**6.97**	**0.19**	**8.16**	**−1.00**
C6	113.5	121.4	−7.9	119.7	−6.2
C1'	123.2	135.2	−12.0	130.6	−7.4
C2'	159.1	172.8	−13.7	173.7	−14.6
C3'	116.3	123.0	−6.7	126.8	−10.5
**H3'**	**7.29**	**7.20**	**0.09**	**7.30**	**−0.01**
C4'	131.5	137.5	−6.0	138.6	−7.1
**H4'**	**7.50**	**7.52**	**−0.02**	**7.46**	**0.04**
C5'	130.5	132.6	−2.1	132.7	−2.2
**H5'**	**7.35**	**7.37**	**−0.02**	**7.39**	**−0.04**
C6'	124.7	138.0	−13.3	136.9	−12.2
**H6'**	**7.23**	**7.31**	**−0.08**	**8.06**	**−0.83**

**RMSD values**		**^13^C NMR = 8.84 ppm** **^1^H NMR = 0.138 ppm**		**^13^C NMR = 8.79 ppm** **^1^H NMR = 0.569 ppm**	

^a^Conformer with Φ = 65 °. ^b^Conformer with Φ = 5 °. ^c^Experimental – theoretical, e.g., C2: 160.9 − 169.1 = −8.2 ppm.

Comparing the experimental and the calculated ^13^C NMR chemical shifts for both the optimized and least-stable DFT-generated conformations, the RMSD values were found to be 8.84 ppm and 8.79 ppm, respectively. The RMSD value for the calculated ^1^H NMR chemical shifts of the optimized conformer was found to be substantially smaller (RMSD = 0.14 ppm) than that for the least-stable conformer (RMSD = 0.57 ppm).

The theoretical coupling constants at the same 24 geometries from geometry scan for coumarin **6** were obtained using the same functional and level of theory (B3LYP/6-311G). The coupling constants (F···C or F···H) for selected nuclei of interest were obtained using a scaling factor calculated from the observed and calculated ^1^*J*_FC_ value for CFCl_3_ [48]. Selected graphs of the plots of *^n^J*_FH_ and *^n^J*_FC_ as a function of rotation of the fluorophenyl ring are included in Supporting Information File 1.

Figures S18 and S19 show the magnitude of ^5^*J*_FH_ and ^6^*J*_FH_ between the fluorine atom and H3 and H5, respectively. It can be seen that, at the B3LYP/6-311G) level, when coupling between F and either H3 or H5 is large enough to be observed in an NMR spectrum (magnitude ≈1–11 Hz for H5, ≈1–5 Hz for H3), coupling to the other nucleus is near zero and may not necessarily be observed. This is consistent with the idea of an average angle in solution that places the fluorine atom closer to H5 than H3, since F–H5 coupling is obvious in the experimental NMR spectrum (Figure 1), while F–H3 coupling is not. A similar prediction is made for coupling between F–C5 and F–C3 (Figures S14 and S17 in Supporting Information File 1). At angles that would manifest in an experimental F–H5 coupling of ≈2.5 Hz, with near-zero F–H3 coupling, Gaussian predicts small F–C coupling to both C5 and C3. This is consistent with the experimental observation of ^5^*J*_FC_ ≈ 1.4 Hz between F and C5 and no observed coupling between F and C3, in the ^13^C-{^1^H} spectrum. Gaussian also predicts F–C coupling between the fluorine atom and carbons C4 and C4a, magnitude <1 Hz and 1–1.5 Hz, respectively. While the <1 Hz F–C4 coupling might not be noticeable in the ^13^C-{^1^H} spectrum, it should be possible to see F–C4a coupling of 1–1.5 Hz, since the F–C5 coupling is observable at a similar magnitude. The model (B3LYP/6-311G) seems to work reasonably well for coupling between F and H3, H5, C3, C4, and C5 but not for C4a.

**Figure 7:**
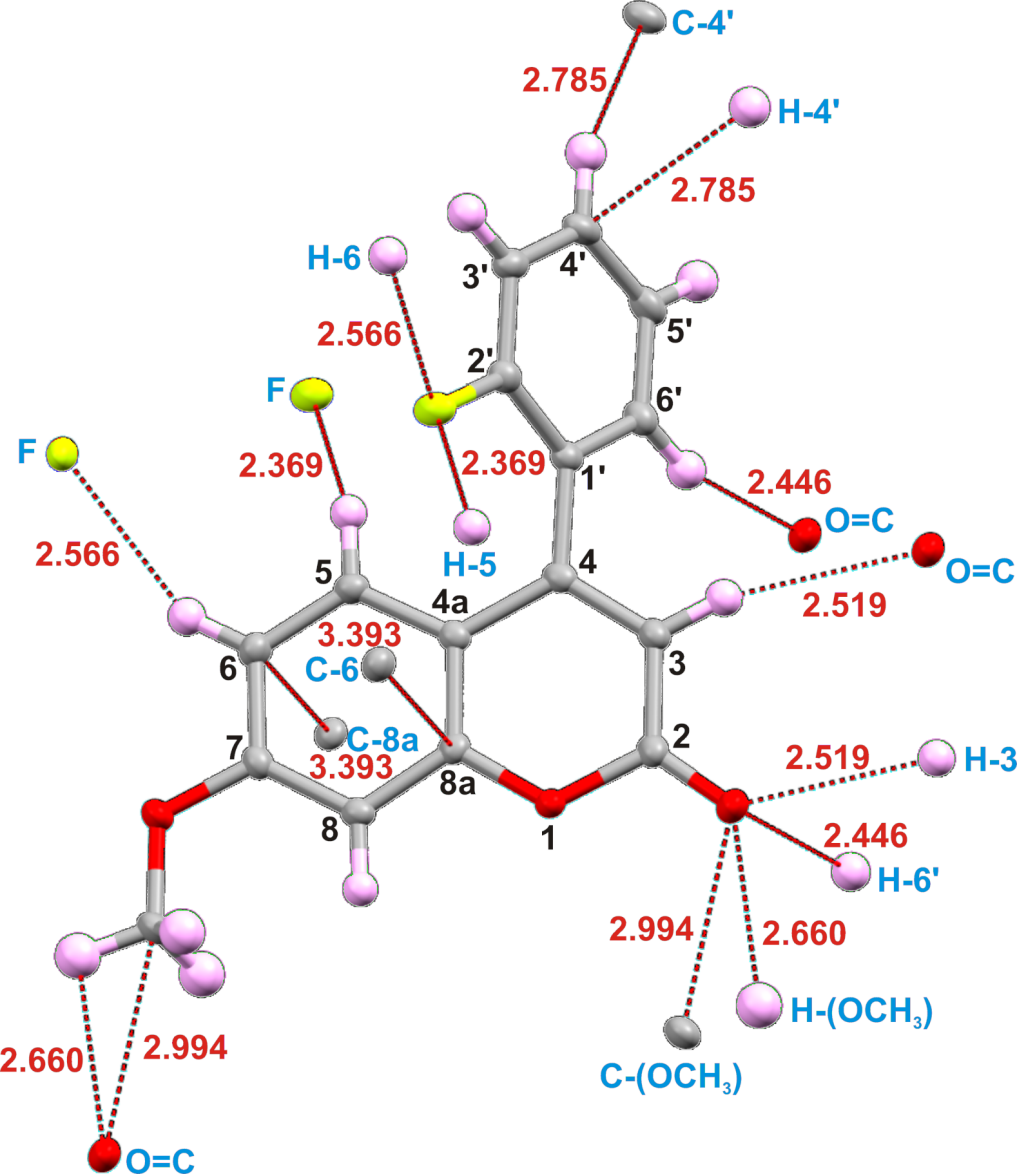
Short contacts within the single-crystal X-ray structure of coumarin **6**.

The single crystal X-ray analysis of coumarin **6** was carried out as it has not been reported previously [CCDC No.: 1868146]. The crystals of **6** were obtained by slow evaporation of methanol/dichloromethane and were found to be of the monoclinic crystal system with space group C2/c (Figure 7).

The crystal structure shows that the fluorinated phenyl ring is at a torsion angle (Φ, C2'–C1'–C4–C4a angle) of 54.44° to the coumarin moiety. The F···H5 TS-distance of 2.547 Å is small enough to induce some rotational constraint on the C4 and C1' bond, as the constraint was observed at an F···H distance of 2.9 Å [37]. The short contact interactions show that there are C–F···H–C intermolecular interactions to the neighboring molecules (F–H6 and F–H5; different molecules) that play a crucial role in crystal packing (Figure 7).

As mentioned above, the structure of coumarin **6** was optimized and the dihedral angle, Φ (C2'–C1'–C4–C4a) was found to be 65.3° (Figure 5), which is comparible close to that found in the crystal structure (Φ = 54.4°). The TS distance between F and H5 for the optimized structure is 2.807 Å which is relatively close to that of the crystal structure (2.547 Å). Selected comparisons are shown in Table 2.

**Table 2:** Comparison of some features of the X-ray crystal structure and DFT optimized structure of **6**: Through-space (TS) and dihedral angle (Φ).

Run	TS distance (Å)			Dihedral angle (°)

	F···H5	F···C5	H6···H3	Φ

**6** (exp)	2.547	2.934	2.535	54.44
**6** (DFT)	2.807	3.174	2.874	65.27
difference	0.260	0.24	0.339	10.83

## Conclusion

The synthesis of 4-(2-fluorophenyl)-7-methoxycoumarin (**6**) via the Pechmann reaction was successful. The solution-state ^1^H and ^13^C NMR spectra of **6** showed that there is a strong intramolecular interaction between F and H5 (*J*_FH_ = 2.6 Hz) and suggest that this interaction is through-space C–F···H–C coupling, since C5 is coupled to F (*J*_FC_ = 1.4 Hz) whereas C4 and C4a are not. The 2D HOESY spectrum shows the F···H5 coupling and also F···H3 coupling, seemingly weaker than F···H5 since splitting of the H3 signal is not observed in the ^1^H and ^1^H-{^19^F} 1D spectra. The single crystal X-ray structure showed that the fluorinated phenyl ring is orientated in a manner that brings the fluorine atom closer to H5 than H3. The same orientation was observed in the DFT-optimized (B3LYP/6-311G) structure. The X-ray data also showed the intermolecular C–F···H–C interactions which, together with other interactions, are resposible for the crystal packing.

## Experimental

### General

All reagents (including solvents) were purchased from the chemical suppliers Aldrich, Fluka and Merck. For all moisture-sensitive reactions, the glassware was thoroughly dried in an oven at ca. 140 °C for 12 h prior to use, and anhydrous solvents were used under inert conditions. Qualitative thin-layer chromatography (TLC, silica gel 60_254_, aluminum backed) was used to monitor the reactions. Visualization of the TLC plates was achieved using an iodine tank and/or fluorescence on exposure to short wavelength ultraviolet light (254 nm). For purification, column chromatography (silica gel 60, 0.040–0.063 mm) or centrifugal chromatography conducted on a Harrison Research Chromatotron model 7924T (glass plates coated with silica gel 60 PF_254_ containing gypsum, 2 and 4 mm thick layer) was used.

Nuclear magnetic resonance (NMR) spectra were recorded on a Bruker Avance 400 spectrometer equipped with a 5 mm BBOZ probe at frequencies of 400 MHz, 100 MHz, and 376 MHz for ^1^H, ^13^C, and ^19^F, respectively. High-resolution mass spectrometry (HRMS) was performed on a Waters LCT Premier time-of-flight mass spectrometer.

**Synthesis of methyl 2-fluorobenzoylacetate (3):** To a stirred mixture of MgCl_2_ (2.0 g, 21 mmol) and Et_3_N (2.1 g, 21 mmol) in dry DCM (15 mL) at room temperature, methyl acetoacetate (**2**, 2.0 g, 17 mmol) was added slowly. The mixture was stirred for 30 min before the temperature was reduced to 0 °C. *n*-BuLi (20 mL of a 1.6 M in hexane, 32 mmol) was added slowly into the mixture and the mixture was stirred for a further 30 min. 2-Fluorobenzoyl chloride (**1**, 2.7 g, 17 mmol) was added dropwise into the mixture and the mixture was stirred for 15 min. The reaction mixture was allowed to reach room temperature and was stirred overnight. To the reaction, was added 5 M HCl (8 mL) and distilled water (10 mL) and the mixture was extracted with DCM (3 × 30 mL). The organic layer was dried over anhydrous MgSO_4_ and the solvent was removed in vacuo. The resulting yellow product was purified by silica gel column chromatography with 10% EtOAc in hexane as eluent and **3** was obtained as a light orange viscous liquid (2.7 g, 81%), TLC *R*_f_ 0.50 (hexane/EtOAc, 9:1). ^1^H NMR (400 MHz, CDCl_3_) 3.76 (s, 3H, H4), 4.01 (d, ^1h^*J*_H,F_ = 3.4 Hz, 2H, H2), 7.15 (ddd, ^3^*J*_H,F_ = 12.1 Hz, ^3^*J*_H,H_ = 8.5 Hz, ^4^*J*_H,H_ = 1.0 Hz, 1H, H3'), 7.26 (t, ^3^*J*_H,H_ = 7.6 Hz, 1H, H5'), 7.57 (m, 1H, H4'), 7.95 (ddd, ^3^*J*_H,H_ = 7.6 Hz, ^4^*J*_H,F_ = 6.2 Hz, ^4^*J*_H,H_ = 1.9 Hz, 1H, H6'); ^13^C NMR (100 MHz, CDCl_3_) 49.6 (d, ^2h^*J*_C,F_ = 8.1 Hz, C2), 52.3 (C4), 116.7 (d, ^2^*J*_C,F_ = 24.1 Hz, C3'), 124.7 (d, ^3^*J*_C,F_ = 2.9 Hz, C6'), 129.3 (d, ^2^*J*_C,F_ = 21.7 Hz, C1'), 131.0 (d, ^4^*J*_C,F_ = 2.3 Hz, C5'), 135.5 (d, ^3^*J*_C,F_ = 9.6 Hz, C4'), 162.2 (d, ^1^*J*_C,F_ = 254.3 Hz, C2’), 167.8 (d, ^3h^*J*_C,F_ = 3.0 Hz, C3), 190.1 (d, ^TS^*J*_C,F_ = 3.7 Hz, C1).

**Synthesis of 7-hydroxy-4-(2-fluorophenyl)coumarin (5):** To a mixture of resorcinol (2.0 g, 18 mmol) and methyl 2-fluorobenzoylacetate (3.5 g, 18 mmol) was added H_2_SO_4_ (8 mL, 75%). The temperature of a stirred mixture was increased to 35 °C. After stirring for 5 h, the mixture was poured into crushed ice and neutralized with a NaOH solution. The mixture was filtered under vacuum and the residue was washed with plenty of water. The resulting product was purified by silica gel column chromatography with 60% EtOAc in hexane as eluent and **5** was obtained as a light yellow solid (4.2 g, 91%), mp 204–207 °C, TLC *R*_f_ 0.45 (hexane/EtOAc, 2:3). ^1^H NMR (400 MHz, DMSO-*d*_6_) 6.24 (s, 1H, H3), 6.77 (dd, ^3^*J*_H,H_ = 8.6 Hz, ^4^*J*_H,H_ = 2.4 Hz, 1H, H6), 6.81 (d, ^4^*J*_H,H_ = 2.4 Hz, 1H, H8), 7.03 (dd, ^3^*J*_H,H_ = 8.6 Hz, ^1h^*J*_H,F_ = 2.6 Hz, 1H, H5), 7.37–7.45 (m, 2H, H3', H6'), 7.50 (td, ^3^*J*_H,H_ = 7.5 Hz, ^4^*J*_H,H_ = 1.8 Hz, 1H, H5'), 7.61 (m, 1H, H4'), 10.67 (s, 1H, OH); ^13^C NMR (100 MHz, DMSO-*d*_6_) 102.6 (C8), 110.7 (C4a), 112.1 (C3), 113.4 (C6), 116.1 (d, ^2^*J*_C,F_ = 21.3 Hz, C3'), 122.7 (d, ^2^*J*_C,F_ = 15.3 Hz, C1'), 125.2 (d, ^3^*J*_C,F_ = 3.6 Hz, C6'), 127.9 (d, ^2h^*J*_C,F_ = 1.6 Hz, C5), 130.8 (d, ^4^*J*_C,F_ = 2.9 Hz, C5'), 132.0 (d, ^3^*J*_C,F_ = 8.2 Hz, C4'), 150.3 (C4), 155.2 (C8a), 158.6 (d, ^1^*J*_C,F_ = 248.6 Hz, C2'), 160.0 (C2), 161.6 (C7); HRMS–ESI^+^ (*m*/*z*): [M + Na]^+^ calcd for C_15_H_9_O_3_FNa, 279.0433; found, 279.0437,

**Synthesis of 4-(2-fluorophenyl)-7-methoxycoumarin (6):** A mixture of 7-hydroxy-4-(2-fluorophenyl)coumarin (**5** ,0.77 g, 3.0 mmol), dimethyl sulfate (0.76 g, 6.0 mmol) and K_2_CO_3_ (0.83 g, 6.0 mmol) was refluxed in acetone (20 mL) for 4 h. The reaction mixture was cooled to room temperature and and brine (50 mL) was added then extracted with ethyl acetate (3 × 40 mL). The organic layer was dried over anhydrous MgSO_4_ and the solvent was removed in vacuo. The resulting light yellow product was purified by silica gel column chromatography with 60% EtOAc in hexane as eluent and **6** was obtained as a yellow crystalline solid (0.78 g, 2.9 mmol, 97%), mp 167–170 ºC, TLC *R*_f_ 0.54 (hexanes/EtOAc, 3:2). ^1^H NMR (400 MHz, CDCl_3_) 3.88 (s, 3H, OMe), 6.25 (s, 1H, H3), 6.79 (dd, ^3^*J*_H,H_ = 8.9 Hz, ^4^*J*_H,H_ = 2.5 Hz, 1H, H6), 6.89 (d, ^4^*J*_H,H_ = 2.5 Hz, 1H, H8), 7.16 (dd, ^3^*J*_H,H_ = 8.9 Hz, ^1h^*J*_H,F_ = 2.5 Hz, 1H, H5), 7.23 (dd, ^3^*J*_H,H_ = 7.8 Hz, ^4^*J*_H,F_ = 4.5 Hz, 1H, H6'), 7.29 (td, ^3^*J*_H,H_ = ^3^*J*_H,F_ = 7.8 Hz, ^4^*J*_H,H_ = 1.8 Hz, 1H, H3'), 7.35 (td ^3^*J*_H,H_ = 7.8 Hz, ^4^*J*_H,H_ = 1.8 Hz, 1H, H5'), 7.50 (m, 1H, H4'); ^13^C NMR (100 MHz, CDCl_3_) 55.8 (OMe), 101.0 (C8), 112.4 (C4a), 112.5 (C3), 113.5 (C6), 116.3 (d, ^2^*J*_C,F_ = 21.9 Hz, C3'), 123.2 (d, ^2^*J*_C,F_ = 15.4 Hz, C1'), 124.7 (d, ^3^*J*_C,F_ = 3.7 Hz, C6'), 127.8 (d, ^2h^*J*_C,F_ = 1.4 Hz, C5), 130.5 (d, ^4^*J*_C,F_ = 3.1 Hz, C5'), 131.5 (d, ^3^*J*_C,F_ = 7.9 Hz, C4'), 150.5 (C4), 155.7 (C8a), 159.1 (d, ^1^*J*_C,F_ = 250.0 Hz, C2'), 160.9 (C2), 163.0 (C7). HRMS–ESI^+^ (*m*/*z*): [M + Na]^+^ calcd for C_16_H_11_O_3_FNa, 293.0590; found, 293.0587.

## Supporting Information

File 1 Copies of NMR spectra for compound **3**, **5** and **6**, single crystal X-ray data for compound **6**, Gaussian calculation data of J-values for compound **6** and HRMS for compound **6**.
