# Supplementary material for: Synthesis of 4-(2-fluorophenyl)-7-methoxycoumarin: experimental and computational evidence for intramolecular and intermolecular C–F···H–C bonds
Source: Beilstein J Org Chem. 2020 Feb 10;16:190–9. doi: 10.3762/bjoc.16.22 (PMC7034221; doi:10.3762/bjoc.16.22)

BEILSTEIN JOURNAL OF ORGANIC CHEMISTRY

Supporting Information

for

Synthesis of 4-(2-fluorophenyl)-7-methoxycoumarin: experimental and computational evidence for intramolecular and intermolecular C–F···H–C bonds

Vuyisa Mzozoyana, Fanie R. van Heerden and Craig Grimmer

Beilstein J. Org. Chem. **2020,** *16,* 190–199. doi:10.3762/bjoc.16.22

Copies of NMR spectra for compound 3, 5 and 6, single crystal X-ray data for compound 6, Gaussian calculation data of J-values for compound 6 and HRMS for compound 6

License and Terms: This is a supporting information file under the terms of the Creative Commons Attribution License (https://creativecommons.org/licenses/by/4.0). Please note that the reuse, redistribution and reproduction in particular requires that the authors and source are credited.
The license is subject to the *Beilstein Journal of Organic Chemistry* terms and conditions: (https://www.beilstein-journals.org/bjoc)

Table of content:

NMR spectra for compound **3**, **5** and **6**……………………………………………………S2

Single crystal X-ray data for compound **6**…………………………………………………S11

Gaussian calculation data of J-values for compound **6**………………………………….S21

HRMS for compound **6** ……………………………………………………………………..S25

Figure S1: ^{1}H NMR spectra for methyl 2-fluorobenzoylacetate (**3**) in $CDCl_3$.

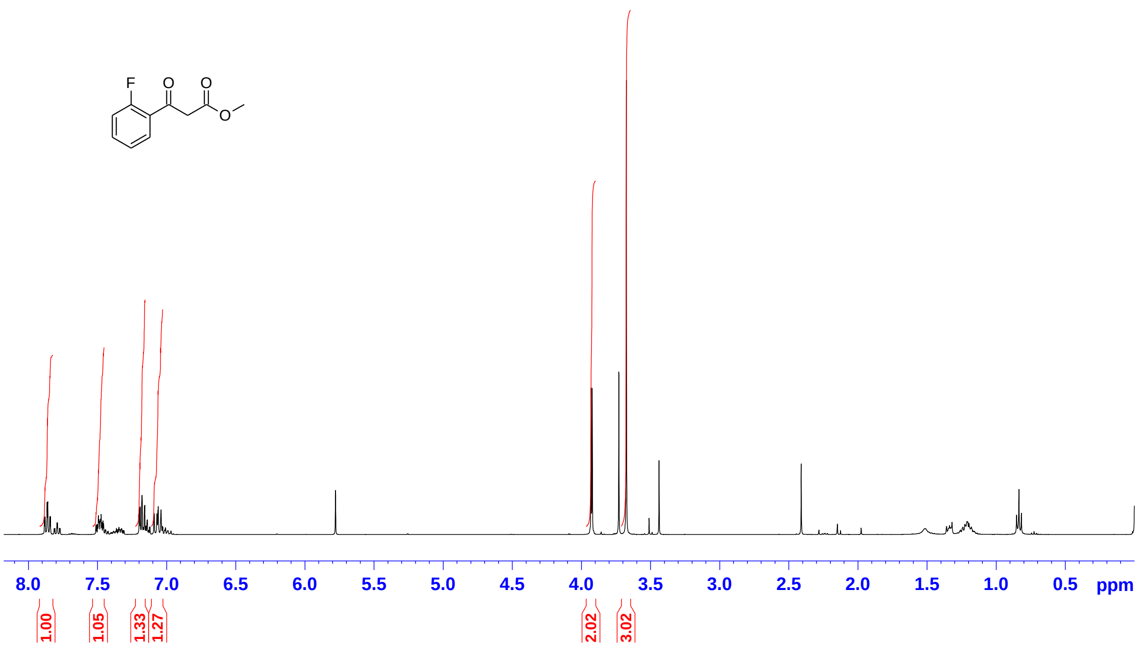

Figure S2: ^{13}C NMR spectra for methyl 2-fluorobenzoylacetate (**3**) in $CDCl_3$.

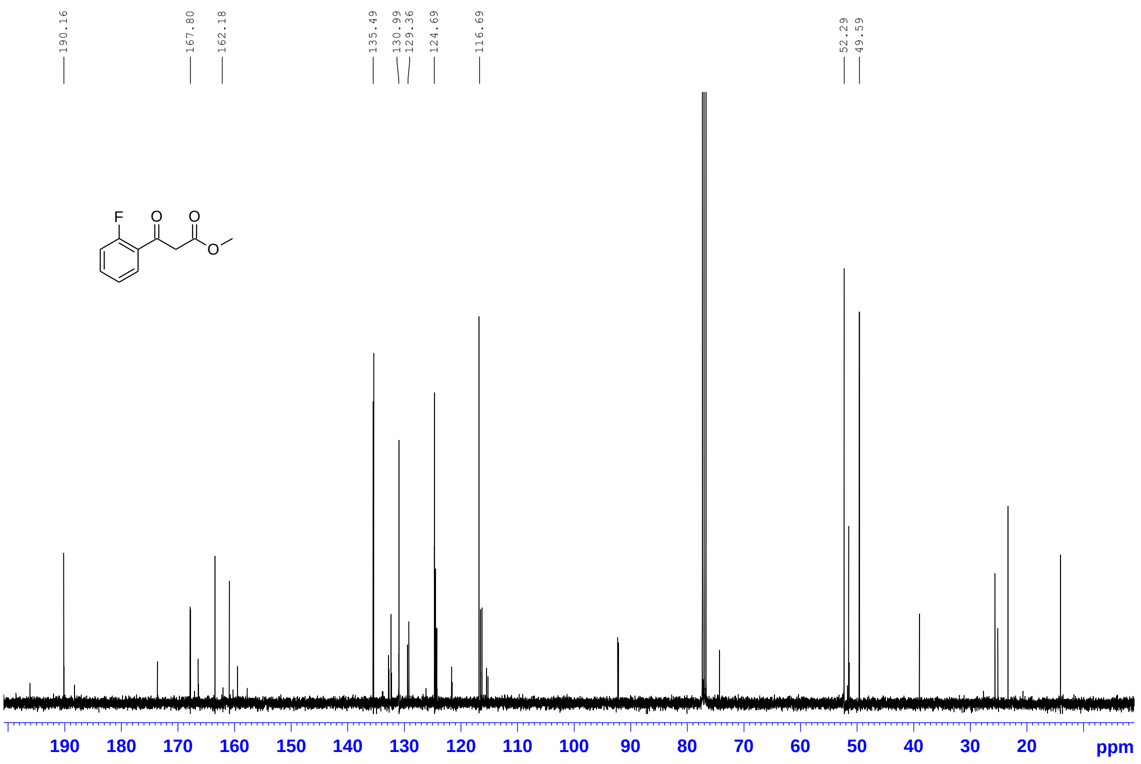

Figure S3: ^{1}H NMR spectra for 7-hydroxy-4-(2-fluorophenyl)coumarin (**5**) in DMSO-d_6.

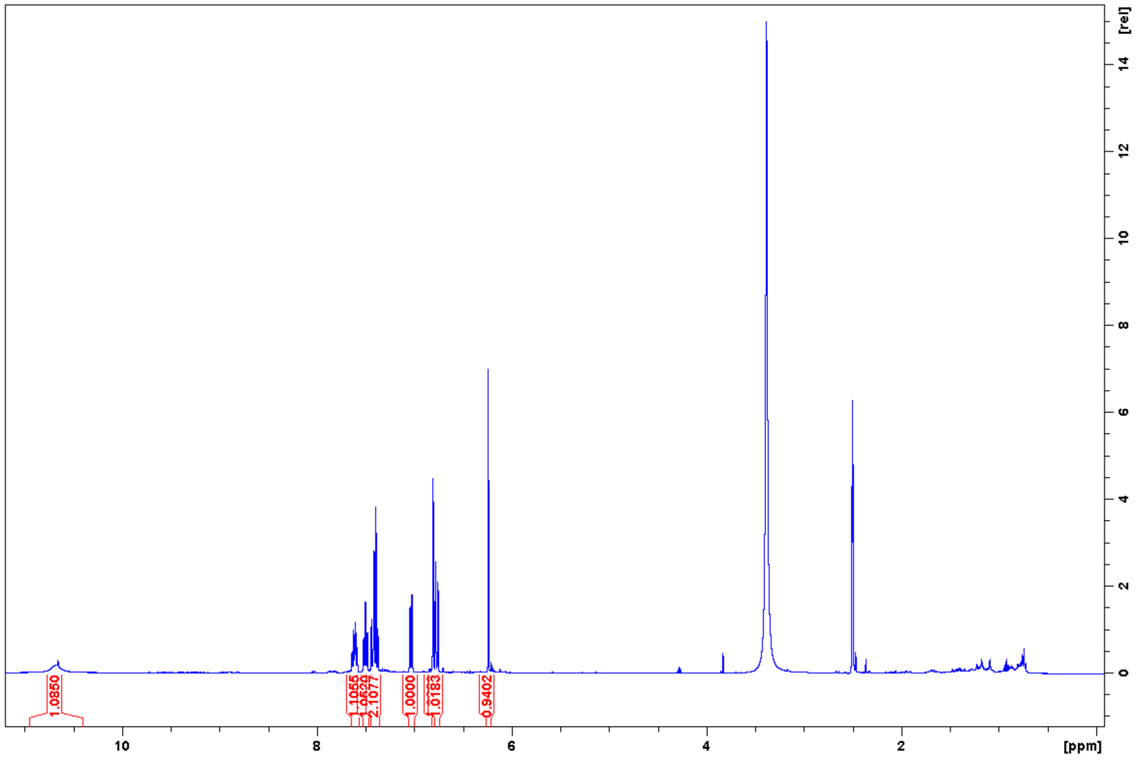

Figure S4: ^{13}C NMR spectra for 7-hydroxy-4-(2-fluorophenyl)coumarin (**5**) in DMSO-d_6.

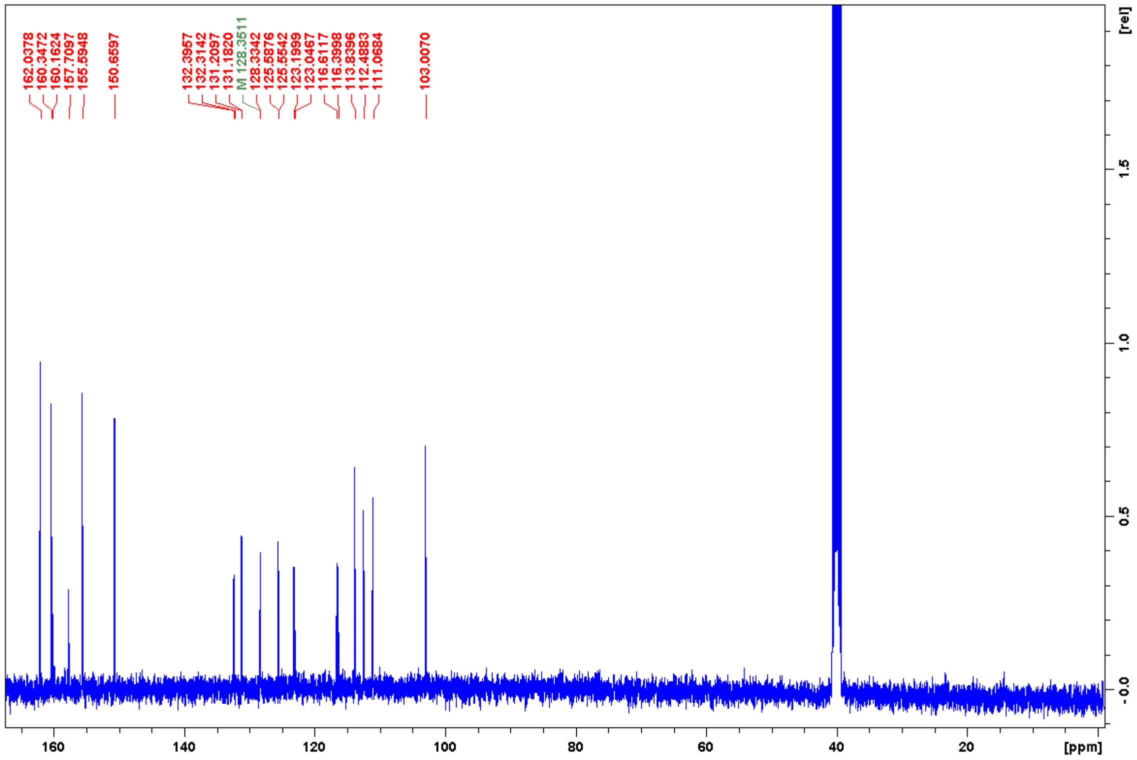

Figure S5: ^{1}H NMR spectra for 4-(2-fluorophenyl)-7-methoxycoumarin (**6**) in $CDCl_3$.

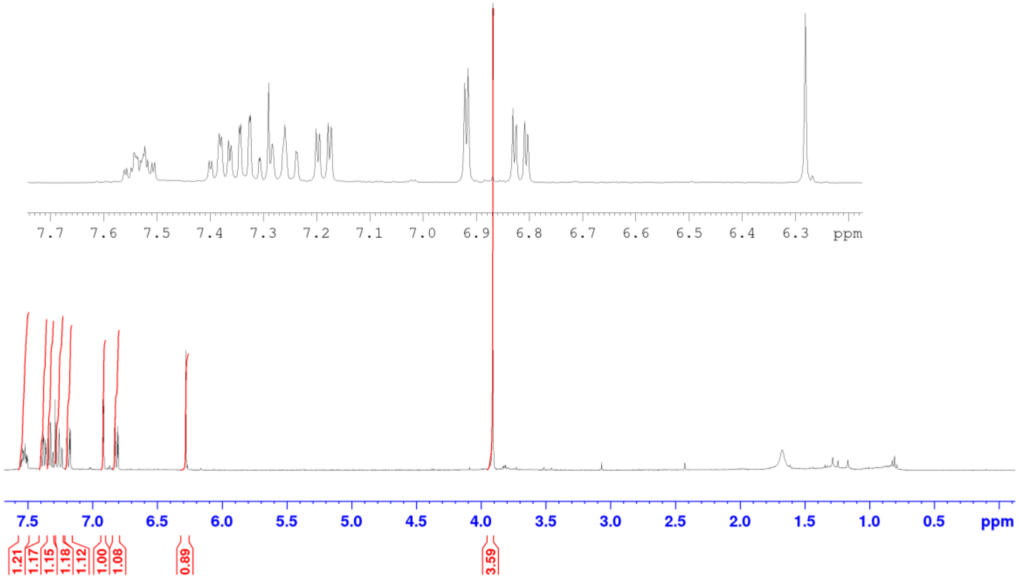

Figure S6: ^{13}C NMR spectra for 4-(2-fluorophenyl)-7-methoxycoumarin (**6**) in $CDCl_3$.

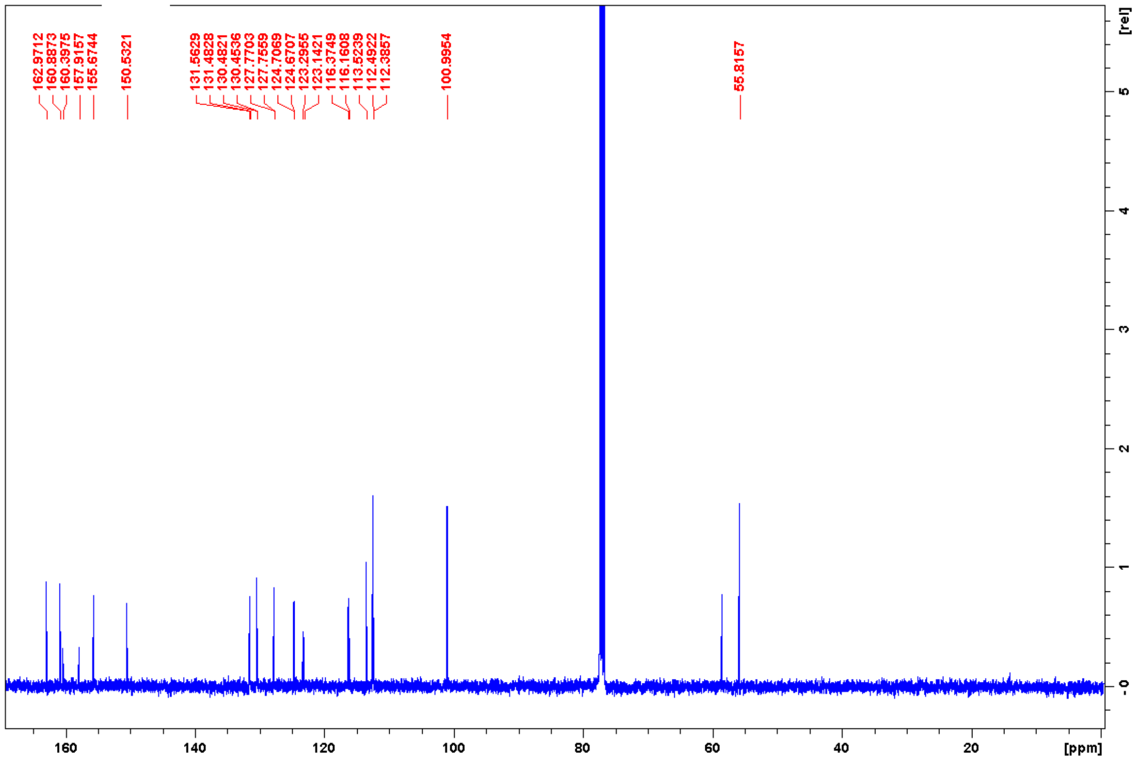

Figure S7: ^{19}F NMR spectrum for 4-(2-fluorophenyl)-7-methoxycoumarin (**6**) in $CDCl_3$.

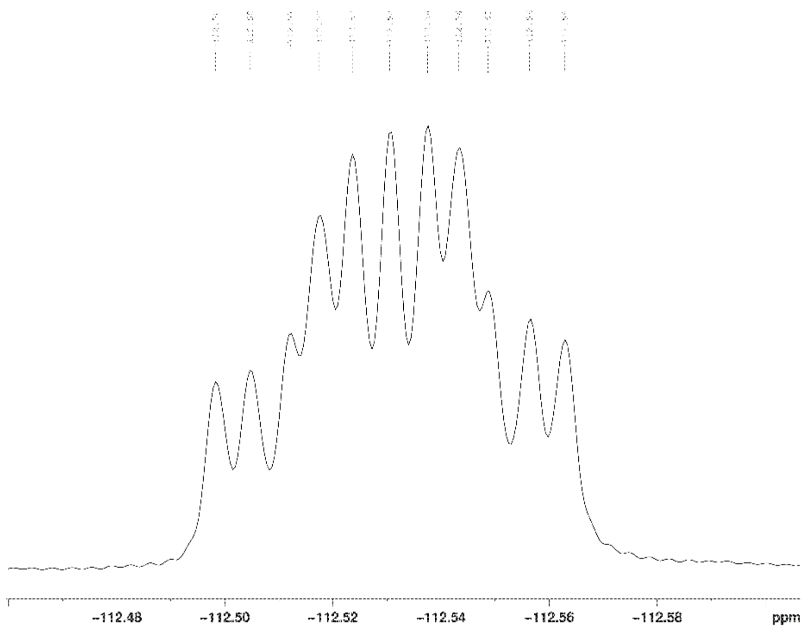

Figure S8: ^{19}F-{^{1}H} NMR spectrum for 4-(2-fluorophenyl)-7-methoxycoumarin (**6**) in $CDCl_3$.

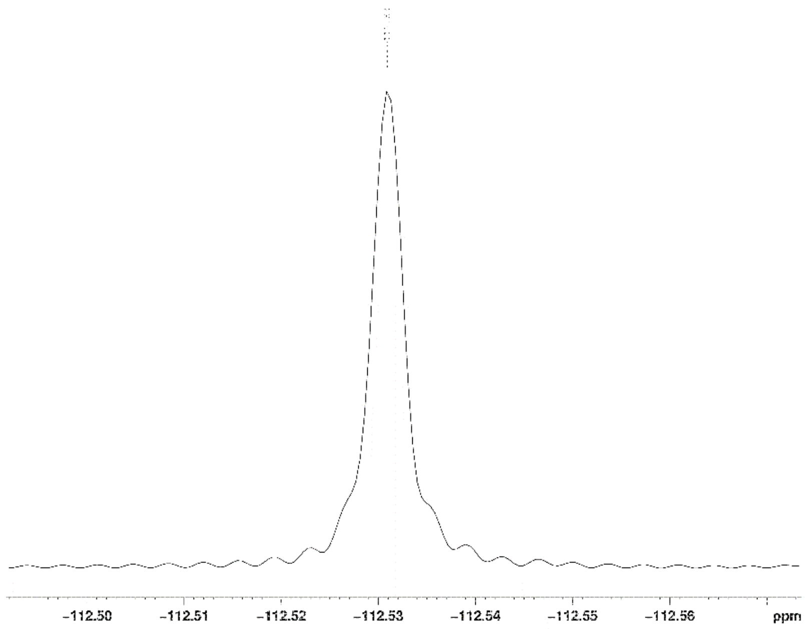

Figure S9: COSY NMR spectrum for 4-(2-fluorophenyl)-7-methoxycoumarin (**6**).

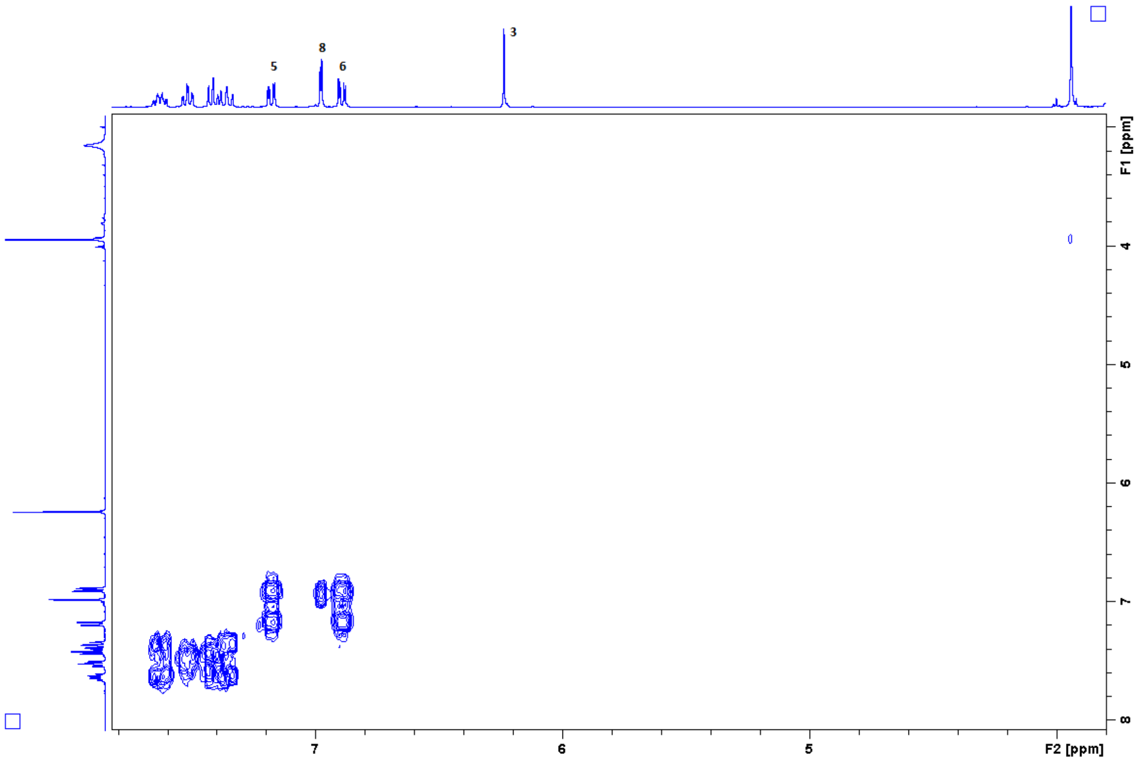

Figure S10a: Compound **6**: comparison of the aromatic region for ^1H and ^1H-{^{19}F} spectra in $CDCl_3$ and acetone-d_6.

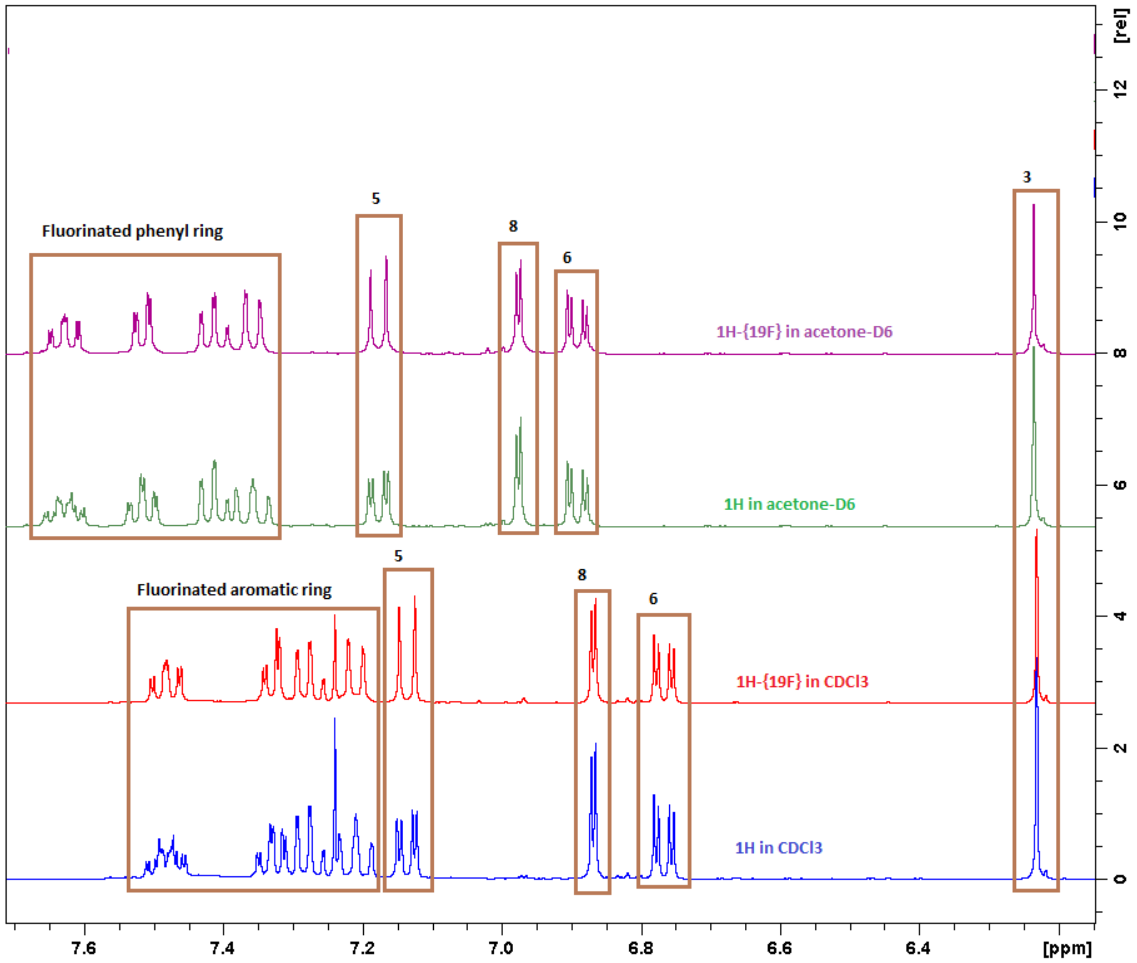

Figure S10b: Compound **5**: comparison of the aromatic region for ^{1}H and ^{1}H-{^{19}F} spectra in CD_3OD and acetone-d_6.

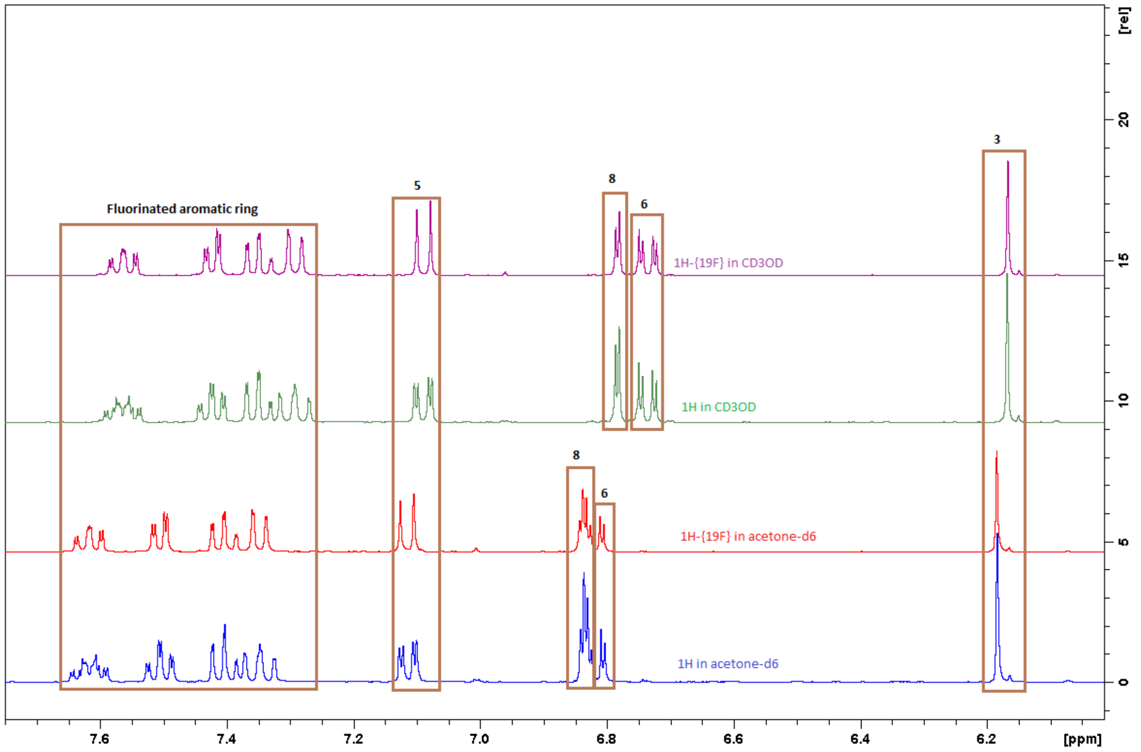

Figure S11: Single crystal X-ray data for compound **6**.

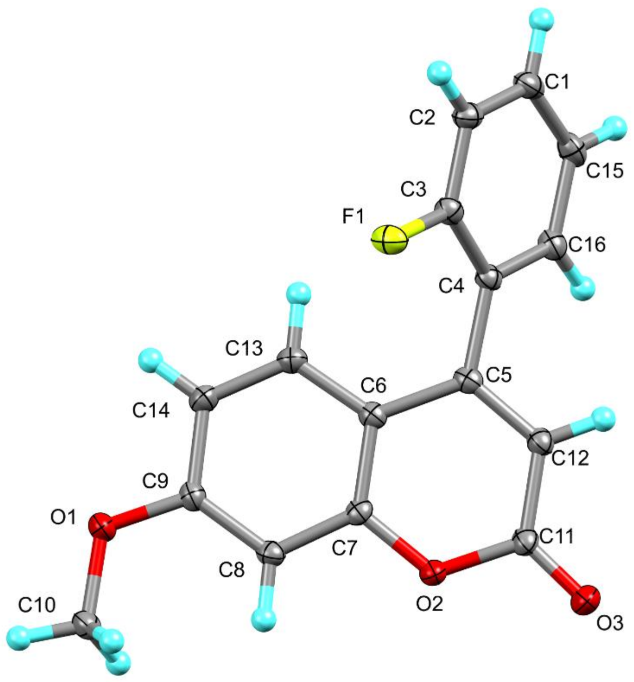

Table S1. Crystal data and structure refinement for **6**.

Identification code	shelx	
Empirical formula	C16 H11 F O3	
Formula weight	270.25	
Temperature	100(2) K	
Wavelength	0.71073 Å	
Crystal system	Monoclinic	
Space group	C 2/c	
Unit cell dimensions	a = 24.3682(12) Å	α= 90°.
	b = 4.0911(2) Å	β= 114.677(2)°.
	c = 26.7036(13) Å	γ = 90°.
Volume	2419.0(2) Å^3	
Z	8	
Density (calculated)	1.484 Mg/m^3	
Absorption coefficient	0.112 mm^{-1}	
F(000)	1120	
Crystal size	0.250 x 0.220 x 0.050 mm^3	
Theta range for data collection	1.678 to 28.535°.	
Index ranges	-32<=h<=32, -5<=k<=4, -35<=l<=35	
Reflections collected	14829	
Independent reflections	3060 [R(int) = 0.0168]	
Completeness to theta = 25.242°	99.7 %	
Absorption correction	Semi-empirical from equivalents	
Max. and min. transmission	0.998 and 0.962	
Refinement method	Full-matrix least-squares on F^2	
Data / restraints / parameters	3060 / 0 / 182	
Goodness-of-fit on F^2	1.080	
Final R indices [I>2sigma(I)]	R1 = 0.0394, wR2 = 0.1081	
R indices (all data)	R1 = 0.0442, wR2 = 0.1122	
Extinction coefficient	n/a	
Largest diff. peak and hole	0.369 and -0.223 e.Å^{-3}	

Table S2. Atomic coordinates (x 10^4) and equivalent isotropic displacement parameters (Å^2× 10^3) for **6**. U(eq) is defined as one third of the trace of the orthogonalized U^{ij} tensor.

	x	y	z	U(eq)
F1	741(1)	9677(2)	7783(1)	22(1)
O1	-904(1)	4183(3)	8763(1)	21(1)
O2	951(1)	9882(2)	9721(1)	17(1)
O3	1806(1)	12469(3)	10200(1)	24(1)
C1	2035(1)	5839(3)	7710(1)	21(1)
C2	1484(1)	7410(3)	7550(1)	19(1)
C3	1280(1)	8105(3)	7948(1)	17(1)
C4	1599(1)	7335(3)	8503(1)	16(1)
C5	1372(1)	8136(3)	8928(1)	15(1)
C6	780(1)	7089(3)	8870(1)	15(1)
C7	592(1)	8000(3)	9277(1)	15(1)
C8	36(1)	7130(3)	9265(1)	16(1)
C9	-347(1)	5203(3)	8829(1)	16(1)
C10	-1122(1)	5329(4)	9154(1)	21(1)
C11	1525(1)	10813(3)	9797(1)	18(1)
C12	1730(1)	9825(3)	9384(1)	17(1)
C13	382(1)	5113(3)	8440(1)	17(1)
C14	-167(1)	4175(3)	8419(1)	18(1)
C15	2376(1)	5086(4)	8260(1)	22(1)
C16	2161(1)	5847(3)	8654(1)	19(1)

Table S3. Bond lengths [Å] and angles [°] for **6**.

F1-C3	1.3590(14)
O1-C9	1.3595(14)
O1-C10	1.4339(15)
O2-C7	1.3773(15)
O2-C11	1.3799(15)
O3-C11	1.2118(16)
C1-C2	1.3858(18)
C1-C15	1.3885(19)
C1-H1	0.9500
C2-C3	1.3792(16)
C2-H9	0.9500
C3-C4	1.3925(17)
C4-C16	1.3966(17)
C4-C5	1.4898(16)
C5-C12	1.3556(17)
C5-C6	1.4495(16)
C6-C7	1.3945(16)
C6-C13	1.4080(17)
C7-C8	1.3884(17)
C8-C9	1.3923(17)
C8-H4	0.9500
C9-C14	1.4037(17)
C10-H2	0.9800
C10-H11	0.9800
C10-H3	0.9800
C11-C12	1.4463(17)
C12-H10	0.9500
C13-C14	1.3705(17)
C13-H5	0.9500
C14-H6	0.9500
C15-C16	1.3901(18)
C15-H8	0.9500
C16-H7	0.9500

C9-O1-C10	117.37(10)
C7-O2-C11	121.47(10)
C2-C1-C15	120.17(12)
C2-C1-H1	119.9
C15-C1-H1	119.9
C3-C2-C1	118.31(12)
C3-C2-H9	120.8
C1-C2-H9	120.8
F1-C3-C2	117.25(11)
F1-C3-C4	119.13(11)
C2-C3-C4	123.61(11)
C3-C4-C16	116.67(11)
C3-C4-C5	122.72(11)
C16-C4-C5	120.58(11)
C12-C5-C6	119.04(11)
C12-C5-C4	119.41(11)
C6-C5-C4	121.54(11)
C7-C6-C13	116.79(11)
C7-C6-C5	118.32(11)
C13-C6-C5	124.86(11)
O2-C7-C8	115.28(10)
O2-C7-C6	121.49(11)
C8-C7-C6	123.23(11)
C7-C8-C9	118.15(11)
C7-C8-H4	120.9
C9-C8-H4	120.9
O1-C9-C8	124.46(11)
O1-C9-C14	115.35(11)
C8-C9-C14	120.20(11)
O1-C10-H2	109.5
O1-C10-H11	109.5
H2-C10-H11	109.5
O1-C10-H3	109.5
H2-C10-H3	109.5
H11-C10-H3	109.5
O3-C11-O2	116.65(11)

O3-C11-C12	125.79(12)
O2-C11-C12	117.53(11)
C5-C12-C11	121.98(11)
C5-C12-H10	119.0
C11-C12-H10	119.0
C14-C13-C6	121.48(11)
C14-C13-H5	119.3
C6-C13-H5	119.3
C13-C14-C9	120.13(12)
C13-C14-H6	119.9
C9-C14-H6	119.9
C1-C15-C16	120.22(12)
C1-C15-H8	119.9
C16-C15-H8	119.9
C15-C16-C4	120.97(12)
C15-C16-H7	119.5
C4-C16-H7	119.5

Table S4. Anisotropic displacement parameters (Å^2× 10^3) for **6**. The anisotropic displacement factor exponent takes the form: $-2\pi^2$[$h^2a^{*2}U^{11}$ + ... + 2 h k a* b* U^{12}].

	U^{11}	U^{22}	U^{33}	U^{23}	U^{13}	U^{12}
F1	21(1)	27(1)	18(1)	4(1)	8(1)	10(1)
O1	17(1)	27(1)	20(1)	-4(1)	10(1)	-5(1)
O2	15(1)	22(1)	13(1)	-2(1)	6(1)	-2(1)
O3	20(1)	34(1)	18(1)	-7(1)	7(1)	-6(1)
C1	23(1)	21(1)	25(1)	-3(1)	16(1)	-3(1)
C2	23(1)	19(1)	17(1)	-1(1)	11(1)	-2(1)
C3	17(1)	15(1)	19(1)	0(1)	9(1)	1(1)
C4	17(1)	15(1)	16(1)	0(1)	8(1)	-1(1)
C5	16(1)	16(1)	15(1)	4(1)	7(1)	3(1)
C6	15(1)	15(1)	15(1)	3(1)	7(1)	2(1)
C7	16(1)	15(1)	13(1)	2(1)	4(1)	2(1)
C8	17(1)	18(1)	14(1)	2(1)	8(1)	2(1)
C9	14(1)	17(1)	18(1)	2(1)	7(1)	0(1)
C10	18(1)	28(1)	21(1)	0(1)	11(1)	-1(1)
C11	15(1)	22(1)	15(1)	2(1)	5(1)	-1(1)
C12	16(1)	20(1)	17(1)	2(1)	7(1)	0(1)
C13	19(1)	17(1)	16(1)	-1(1)	8(1)	1(1)
C14	19(1)	18(1)	17(1)	-3(1)	7(1)	-2(1)
C15	17(1)	23(1)	29(1)	1(1)	13(1)	2(1)
C16	16(1)	21(1)	21(1)	2(1)	8(1)	0(1)

Table S5. Hydrogen coordinates ($\times 10^4$) and isotropic displacement parameters ($Å^2 \times 10^3$) for **6**.

	x	y	z	U(eq)
H1	2180	5277	7441	25
H9	1253	7994	7176	23
H4	-81	7831	9545	19
H2	-853	4573	9524	32
H11	-1530	4474	9057	32
H3	-1134	7724	9149	32
H10	2129	10377	9434	21
H5	497	4415	8158	20
H6	-426	2826	8126	22
H8	2757	4047	8369	26
H7	2400	5348	9030	23

Table S6. Torsion angles [°] for **6**.

C15-C1-C2-C3	-1.8(2)
C1-C2-C3-F1	179.30(11)
C1-C2-C3-C4	0.5(2)
F1-C3-C4-C16	-177.31(11)
C2-C3-C4-C16	1.5(2)
F1-C3-C4-C5	0.63(19)
C2-C3-C4-C5	179.43(12)
C3-C4-C5-C12	-126.92(14)
C16-C4-C5-C12	50.93(17)
C3-C4-C5-C6	54.41(18)
C16-C4-C5-C6	-127.73(13)
C12-C5-C6-C7	2.93(18)
C4-C5-C6-C7	-178.40(11)
C12-C5-C6-C13	-174.90(12)
C4-C5-C6-C13	3.77(19)
C11-O2-C7-C8	177.36(11)
C11-O2-C7-C6	-3.32(18)
C13-C6-C7-O2	178.87(11)
C5-C6-C7-O2	0.87(18)
C13-C6-C7-C8	-1.86(19)
C5-C6-C7-C8	-179.87(11)
O2-C7-C8-C9	-179.45(11)
C6-C7-C8-C9	1.25(19)
C10-O1-C9-C8	3.59(19)
C10-O1-C9-C14	-176.44(11)
C7-C8-C9-O1	-179.71(12)
C7-C8-C9-C14	0.32(19)
C7-O2-C11-O3	179.97(11)
C7-O2-C11-C12	1.92(17)
C6-C5-C12-C11	-4.37(19)
C4-C5-C12-C11	176.93(12)
O3-C11-C12-C5	-175.85(13)
O2-C11-C12-C5	2.01(19)
C7-C6-C13-C14	0.95(18)

C5-C6-C13-C14	178.81(12)
C6-C13-C14-C9	0.5(2)
O1-C9-C14-C13	178.85(11)
C8-C9-C14-C13	-1.2(2)
C2-C1-C15-C16	1.2(2)
C1-C15-C16-C4	0.9(2)
C3-C4-C16-C15	-2.14(19)
C5-C4-C16-C15	179.88(12)

Figure S12: Plot of $^2J_{FC}$ (Hz) vs dihedral angle 2’-1’-4-4a (°) using B3LYP/6-311G.

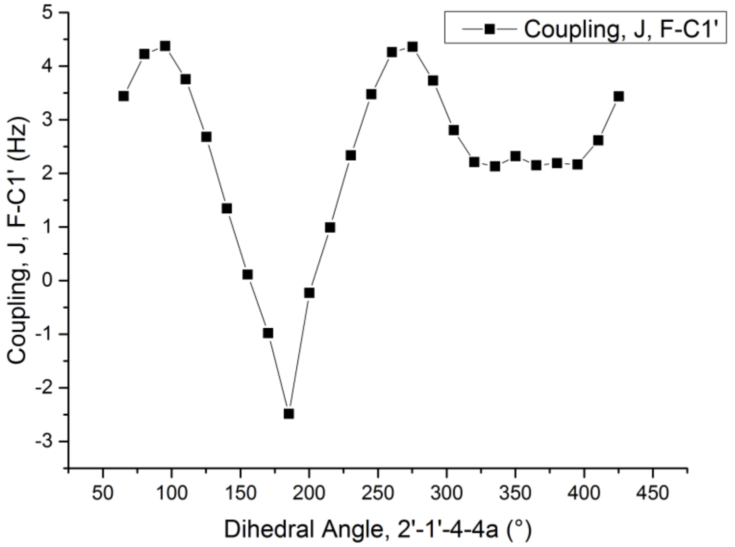

Figure S13: Plot of $^1J_{FC}$ (Hz) vs dihedral angle 2’-1’-4-4a (°) using B3LYP/6-311G.

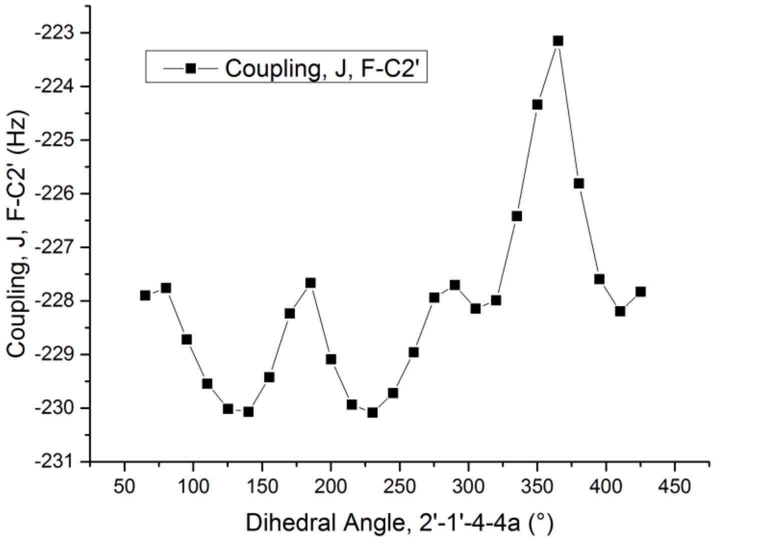

Figure S14: Plot of $^{4}J_{FC}$ (Hz) vs Dihedral Angle 2’-1’-4-4a (°) using B3LYP/6-311G

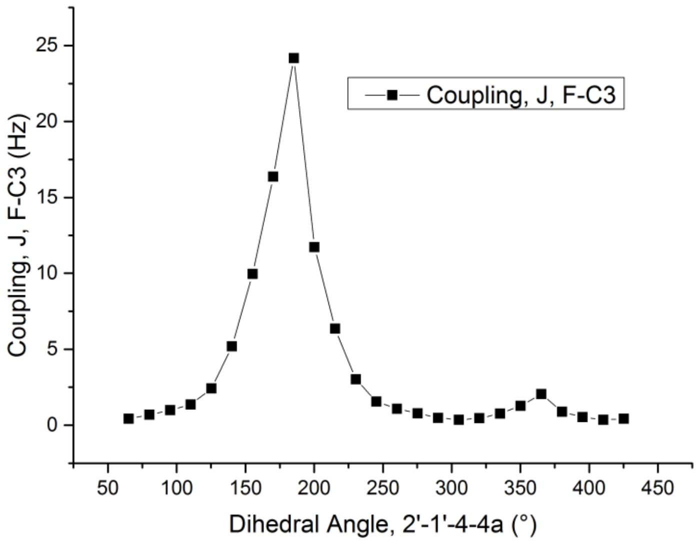

Figure S15: Plot of $^{3}J_{FC}$ (Hz) vs dihedral angle 2’-1’-4-4a (°) using B3LYP/6-311G.

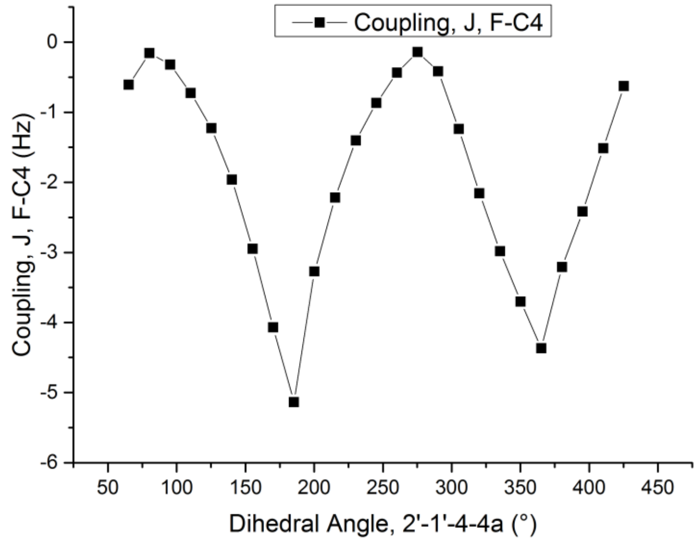

Figure S16: Plot of $^4J_{FC}$ (Hz) vs dihedral angle 2'-1'-4-4a (°) using B3LYP/6-311G.

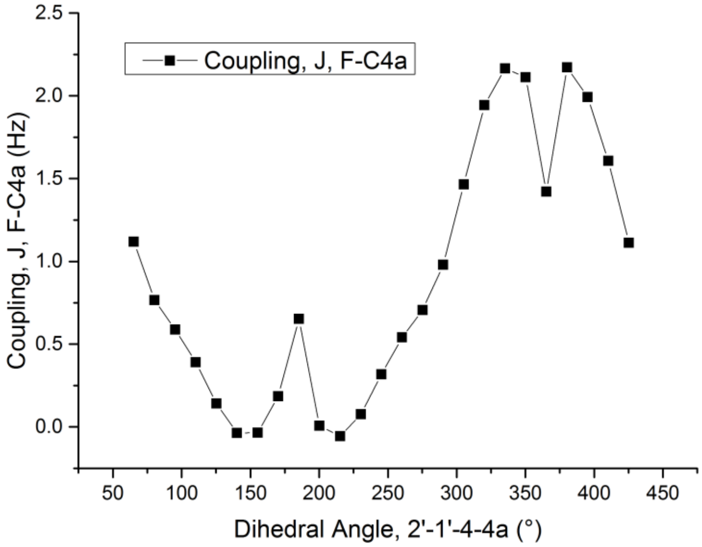

Figure S17: Plot of $^5J_{FC}$ (Hz) vs dihedral angle 2'-1'-4-4a (°) using B3LYP/6-311G.

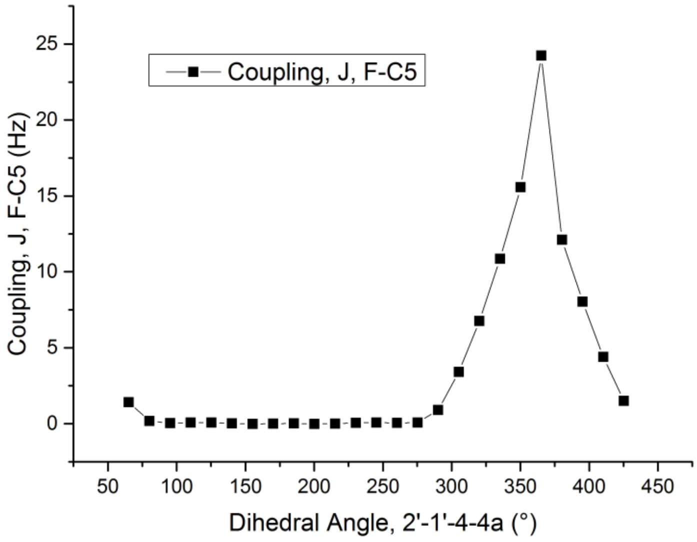

Figure S18: Plot of $^{5}J_{FH}$ (Hz) vs dihedral angle 2'-1'-4-4a (°) using B3LYP/6-311G.

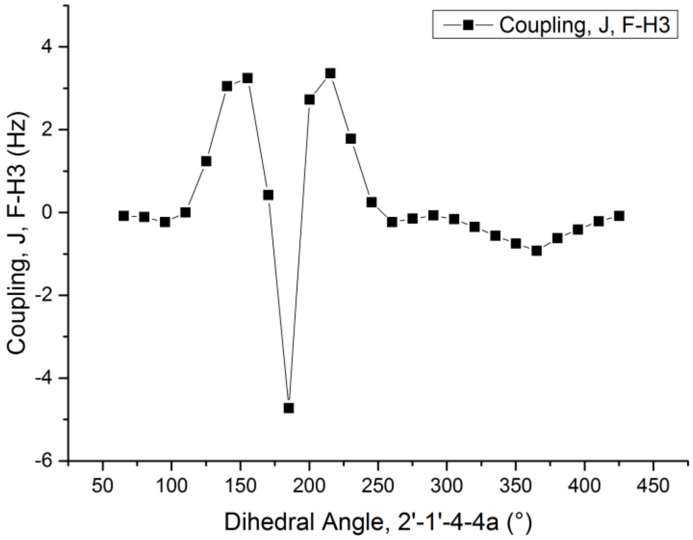

Figure S19: Plot of $^{6}J_{FH}$ (Hz) vs dihedral angle 2'-1'-4-4a (°) using B3LYP/6-311G.

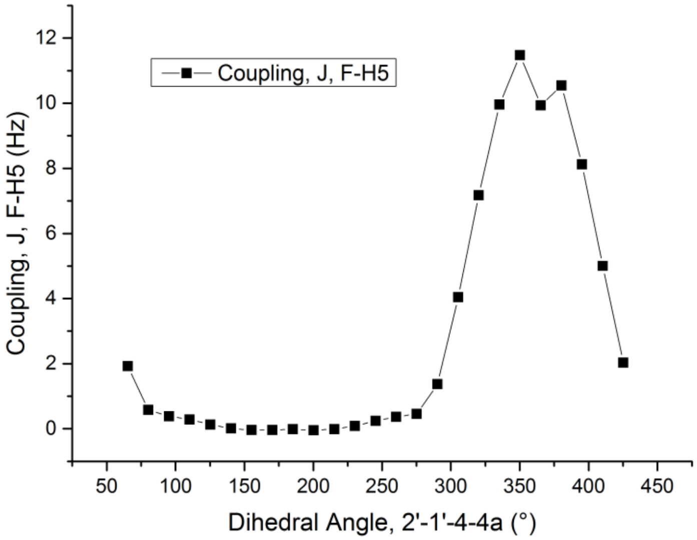

Figure S20: HRMS for compound **6**.

Monoisotopic Mass, Even Electron Ions
7 formula(e) evaluated with 1 results within limits (up to 20 closest results for each mass)
Elements Used:
C: 15-20 H: 10-15 O: 0-5 F: 1-1 Na: 0-1
VMZ-8 32 (1.046) Cm (1:61)
TOF MS ES+

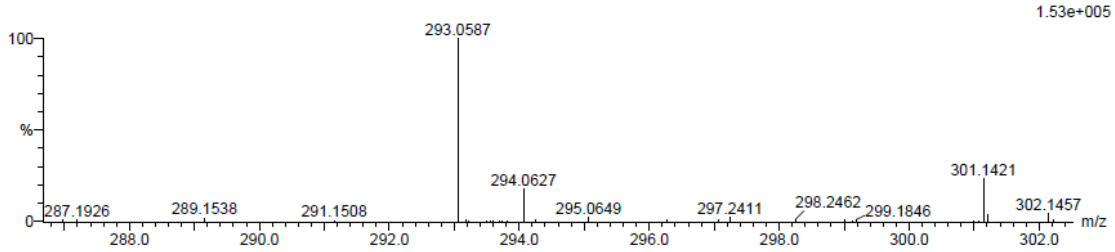

Minimum:				-1.5			
Maximum:		5.0	5.0	100.0			
Mass	Calc. Mass	mDa	PPM	DBE	i-FIT	i-FIT (Norm)	Formula
293.0587	293.0590	-0.3	-1.0	10.5	125.0	0.0	C16 H11 O3 F Na

Figure S21: ^{13}C NMR spectra for coumarin **5** and **6**; to show that there is no splitting of the signal corresponding to C4a.

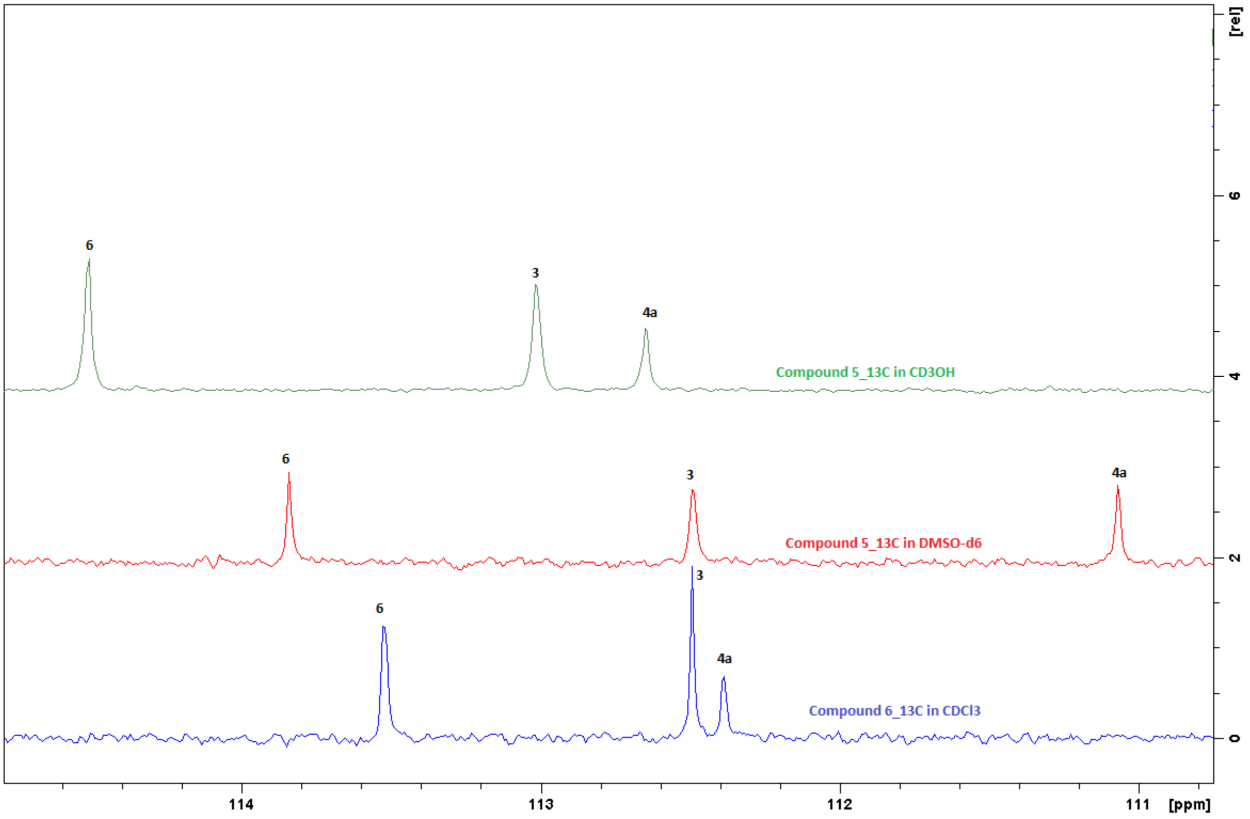

Figure S22: ^{13}C NMR spectra for coumarin **5** and **6**; to show that there is no splitting of the signal corresponding to C4.

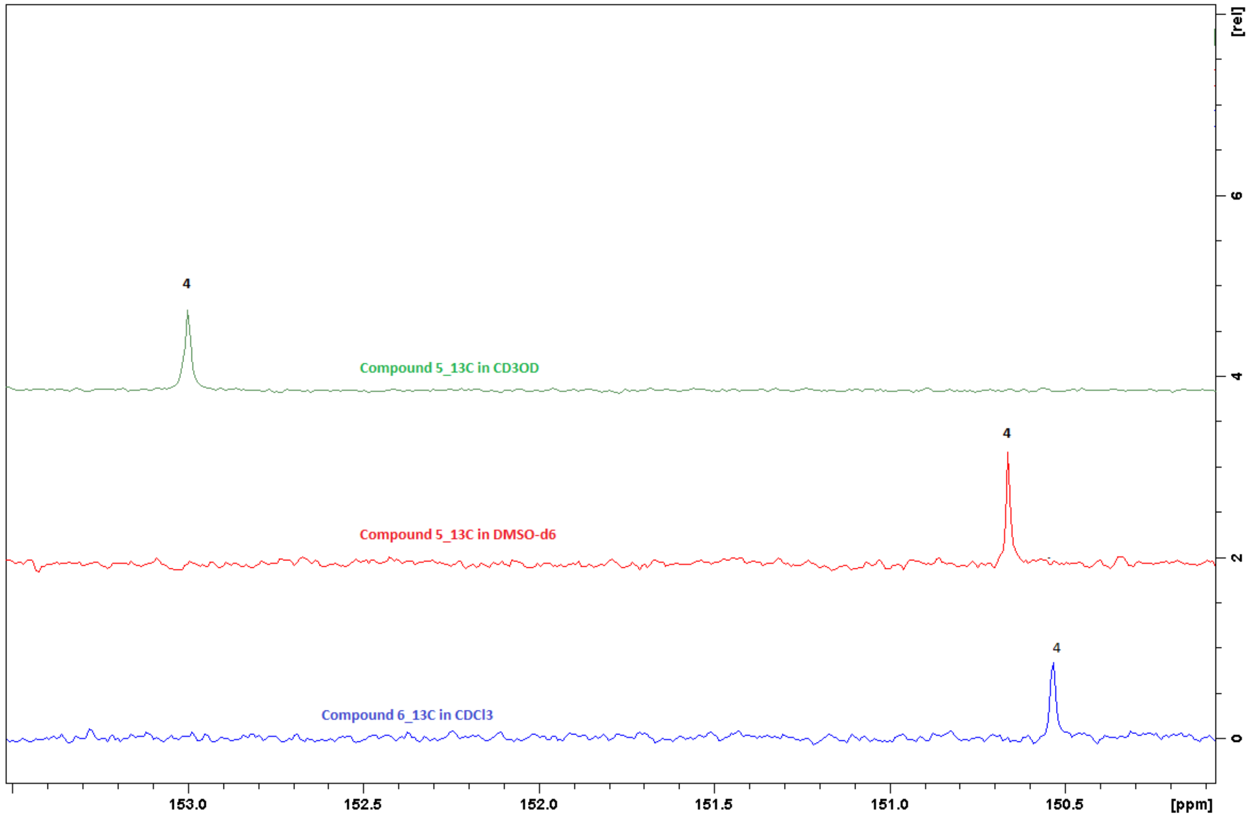

Supplement: File 1 — Copies of NMR spectra for compound 3, 5 and 6, single crystal X-ray data for compound 6, Gaussian calculation data of J-values for compound 6 and HRMS for compound 6. [file Beilstein_J_Org_Chem-16-190-s001.pdf]
